# Enhancing s-CO_2_ Brayton Power Cycle Efficiency in Cold Ambient Conditions Through Working Fluid Blends

**DOI:** 10.3390/e27070744

**Published:** 2025-07-11

**Authors:** Paul Tafur-Escanta, Luis Coco-Enríquez, Robert Valencia-Chapi, Javier Muñoz-Antón

**Affiliations:** 1 Facultad de Ingeniería en Ciencias Aplicadas, Universidad Técnica del Norte, Ibarra 100150, Ecuador; pmtafur@utn.edu.ec (P.T.-E.); rmvalencia@utn.edu.ec (R.V.-C.); 2 Luxoft (DXC Technology), 28232 Madrid, Spain; luiscocoenriquez@hotmail.com; 3 ETSI Industriales, Universidad Politécnica de Madrid, 28006 Madrid, Spain

**Keywords:** Brayton cycle, electrical generation, power systems, s-CO_2_ mixtures

## Abstract

Supercritical carbon dioxide (s-CO_2_) Brayton cycles have emerged as a promising technology for high-efficiency power generation, owing to their compact architecture and favorable thermophysical properties. However, their performance degrades significantly under cold-climate conditions—such as those encountered in Greenland, Russia, Canada, Scandinavia, and Alaska—due to the proximity to the fluid’s critical point. This study investigates the behavior of the recompression Brayton cycle (RBC) under subzero ambient temperatures through the incorporation of low-critical-temperature additives to create CO_2_-based binary mixtures. The working fluids examined include methane (CH_4_), tetrafluoromethane (CF_4_), nitrogen trifluoride (NF_3_), and krypton (Kr). Simulation results show that CH_4_- and CF_4_-rich mixtures can achieve thermal efficiency improvements of up to 10 percentage points over pure CO_2_. NF_3_-containing blends yield solid performance in moderately cold environments, while Kr-based mixtures provide modest but consistent efficiency gains. At low compressor inlet temperatures, the high-temperature recuperator (HTR) becomes the dominant performance-limiting component. Optimal distribution of recuperator conductance (UA) favors increased HTR sizing when mixtures are employed, ensuring effective heat recovery across larger temperature differentials. The study concludes with a comparative exergy analysis between pure CO_2_ and mixture-based cycles in RBC architecture. The findings highlight the potential of custom-tailored working fluids to enhance thermodynamic performance and operational stability of s-CO_2_ power systems under cold-climate conditions.

## 1. Introduction

Supercritical carbon dioxide (s-CO_2_) Brayton power cycles have garnered substantial attention over the past decades as a promising alternative to conventional steam Rankine cycles for power generation [1,2,3]. By operating the working fluid above its critical point (critical temperature ≈ 31 °C, critical pressure ≈ 7.4 MPa), s-CO_2_ cycles can achieve high thermal efficiencies—frequently exceeding 50% at moderate turbine inlet temperatures [1]. Additionally, these cycles exhibit compact turbomachinery and high power density due to the elevated fluid density in the supercritical state [2]. These characteristics make s-CO_2_ systems attractive for diverse applications, including advanced nuclear reactors [3,4,5,6], concentrated solar power (CSP) plants [7,8], and industrial waste heat recovery [9,10]. Notably, the s-CO_2_ Brayton cycle maintains high efficiency even at the intermediate heat source temperatures typical of next-generation nuclear and CSP systems. Furthermore, its single-phase operation enables the potential for reduced capital costs and smaller system footprints compared to conventional water–steam cycles [2,7,9]. The technology has been thoroughly reviewed in the recent literature [2,4,5,6], and several pilot and demonstration initiatives are currently underway to validate its performance under real-world conditions [8].

Despite these advantages, certain operational constraints hinder the broader deployment of s-CO_2_ cycles. As noted by Dostal [10] and further developed by Kulhánek and Dostál [11], various cycle configurations—such as recompression, pre-compression, and partial cooling cycles—have demonstrated thermal efficiencies exceeding 40%, with recompression layouts achieving peak performance near optimal pressure ratios. These advancements underscore the potential of s-CO_2_ systems in future energy conversion technologies that demand a balance of efficiency, modularity, and economic viability.

A primary technical challenge is associated with the thermophysical behavior of CO_2_ near its critical point. The performance of s-CO_2_ cycles is particularly sensitive to the cold-end conditions of the cycle, specifically the temperature and pressure at the cooler and compressor inlet [12,13]. Theoretically, reducing the compressor inlet temperature should enhance the cycle’s pressure ratio and thermal efficiency—analogous to the improved performance observed in Rankine cycles with a colder sink. However, CO_2_’s relatively high critical temperature (~31 °C) limits how far this temperature can be reduced [14]. At or below this threshold, the working fluid approaches two-phase conditions, introducing risks of condensation or unstable operation at the compressor inlet. Consequently, most s-CO_2_ systems are designed to maintain the cooler outlet temperature slightly above the critical point (typically 32–35 °C) to avoid crossing into the two-phase region [14,15,16].

This design constraint becomes particularly problematic in cold environments—such as subfreezing climates or in applications with very low sink temperatures—where the inability to further lower the compressor inlet temperature results in suboptimal cooling and increased compressor work. In contrast to hot climates, where elevated ambient temperatures limit s-CO_2_ cycle efficiency by increasing compressor inlet temperatures [14], extremely low temperatures introduce the inverse problem: phase instability near the critical point constrains the minimum achievable temperature and restricts potential efficiency gains. This limitation highlights a key knowledge gap in adapting s-CO_2_ cycles to cold climates or regions with large seasonal temperature variations.

One promising strategy to overcome this thermodynamic limitation involves modifying the working fluid through binary gas mixtures. Instead of relying on pure CO_2_, the working fluid can be blended with a secondary component to adjust critical properties and improve overall cycle behavior [2,16]. This approach—reminiscent of refrigerant blending in HVAC systems—has precedent in other thermodynamic cycles: helium–xenon mixtures are used in closed Brayton systems to enhance turbomachinery performance [16], while ammonia–water blends in Kalina cycles exploit variable phase behavior for improved heat recovery [16]. In recent years, research has increasingly focused on CO_2_-based mixtures to enhance cycle efficiency or broaden the operational range beyond the limits of pure CO_2_ [17,18]. Comparative analyses and review articles have classified mixture-based strategies as a viable performance-enhancement route alongside other cycle optimization techniques [2,7,16,19].

Early experimental investigations highlighted the trade-offs and advantages of CO_2_ mixtures. For example, Sandia National Laboratories conducted pioneering experiments on a 50 kW s-CO_2_ loop, studying the performance impact of trace amounts of additives such as SF_6_, CH_4_, N_2_, neon, and n-butane [20]. Around the same time, Jeong et al. [4,5,6] proposed adjusting the CO_2_ critical point for better thermodynamic matching with sodium-cooled fast reactors, experimenting with additives including cyclohexane, n-butane, isobutane, propane, and hydrogen sulfide (H_2_S). Among these, the CO_2_/H_2_S mixture yielded the highest thermal efficiency due to H_2_S’s elevated critical temperature (~100 °C), which enabled partial condensation and reduced compression work via liquid-phase pumping in transcritical operation. In parallel, CO_2_/propane mixtures offered enhanced heat transfer and reduced pressure drops in heat exchangers [21], although their gains in overall cycle efficiency were less pronounced. These studies emphasized that additive selection should be tailored based on performance priorities—whether maximizing efficiency, improving heat exchange, or minimizing pressure losses [22,23,24].

Recent research has shifted toward high-critical-temperature additives that promote deliberate condensation at the compressor inlet to enhance efficiency. A notable example is titanium tetrachloride (TiCl_4_), an additive with a critical temperature around 365 °C. Its inclusion in CO_2_ mixtures facilitates transcritical operation resembling a Rankine cycle with liquid-phase pumping. Computational work by Bonalumi et al. [7] and experimental validation by Invernizzi et al. [8] demonstrated that CO_2_–TiCl_4_ mixtures can boost cycle efficiency by up to 5.5% in absolute terms relative to pure CO_2_. Moreover, these heavier blends mitigate high-temperature degradation effects by enabling effective heat rejection at elevated ambient temperatures, thanks to their elevated critical points [18]. These findings further validate working fluid tailoring as a powerful technique for customizing s-CO_2_ cycle behavior to diverse environmental and operational conditions [25,26].

In summary, optimized CO_2_-based mixtures have proven capable of both raising peak efficiencies and expanding operability across wider temperature ranges compared to standard pure-CO_2_ cycles [17,18]. These blends introduce an additional degree of design flexibility, offering potential solutions to challenges that limit pure s-CO_2_ performance in extreme ambient conditions [27,28].

Despite the growing body of research on CO_2_-based mixtures, most studies to date have focused on heavier additives designed for high-temperature or high-ambient applications. These additives—characterized by critical temperatures higher than that of CO_2_—enable cycle configurations involving partial condensation and typically yield efficiency gains when the ambient heat sink is relatively warm or when the objective is to maximize absolute efficiency through recuperation and liquid-phase compression [7,8,18].

In contrast, comparatively limited attention has been devoted to the inverse approach: employing low-critical-temperature additives to enhance cycle performance in cold ambient conditions. Light gases—those with critical temperatures significantly below that of CO_2_—can depress the critical point of the working fluid mixture, theoretically enabling the cycle to remain fully supercritical at lower sink temperatures. This approach offers the potential for higher efficiencies in cold environments by permitting operation with lower compressor inlet temperatures while avoiding two-phase instabilities.

Nonetheless, prior investigations involving light additives have been sparse and somewhat inconclusive. In several cases, the introduction of non-condensable light gases, such as helium or neon, resulted in negligible or slightly adverse effects on cycle efficiency under design-point conditions [18]. For instance, Vesely et al. reported that minor admixtures of low-boiling-point gases (e.g., He, Ar) offered minimal gains in recompression cycle efficiency, with notable effects occurring primarily under off-design scenarios [20]. These results suggest that the efficacy of light-component mixtures likely depends on the extent to which ambient temperatures deviate below the natural critical point of CO_2_ [18]. It has been proposed that the colder the sink temperature relative to 31 °C, the greater the potential efficiency improvement achievable through appropriately formulated low-critical-point mixtures. While this hypothesis is compelling, it remains underexplored. To date, no comprehensive study has systematically optimized s-CO_2_ cycle performance using light additives for operation in extreme cold climates. Most existing research on mixtures has prioritized base-cycle enhancement or high-temperature resilience, thereby leaving a significant knowledge gap regarding cold-weather performance.

This study aims to address that gap by investigating CO_2_-based binary mixtures containing low-critical-temperature additives as a strategy to improve recompression Brayton cycle performance in cold ambient environments. Specifically, four candidate additives were selected—methane (CH_4_), nitrogen trifluoride (NF_3_), carbon tetrafluoride (CF_4_), and krypton (Kr)—due to their critical temperatures being significantly below 0 °C [29,30,31,32].

The incorporation of novel working fluids also raises important considerations related to safety and environmental impact [33]. Previous studies have emphasized the necessity of accounting for such factors when evaluating alternative working fluids [34,35]. In this context, each candidate presents trade-offs. Methane (CH_4_) is flammable and possesses a non-negligible global warming potential (GWP), though its atmospheric lifetime is relatively short. NF_3_ and CF_4_, while chemically stable and inert during operation, are extremely potent greenhouse gases if released—NF_3_ has a 100-year GWP approximately 17,200 times that of CO_2_, and CF_4_ about 7390 times [35,36]. Even minor leaks of these compounds could negate the environmental benefits derived from efficiency gains. In contrast, krypton is chemically inert, non-toxic, and non-flammable, with near-zero direct GWP due to its lack of infrared absorption. However, it is a trace atmospheric component (~1 ppm by volume) and is expensive to obtain in large quantities. Industrial use of Kr would necessitate gas separation infrastructure and introduce economic challenges.

Given these considerations, this study evaluates not only the thermodynamic performance of the proposed CO_2_-based mixtures in cold climates but also assesses their environmental feasibility and operational risks. By jointly examining efficiency improvements and associated environmental costs, the analysis aims to identify which, if any, of these binary mixtures offer a compelling balance between performance enhancement and sustainability. The findings are intended to support the development of s-CO_2_ Brayton systems capable of efficient operation across a broader ambient temperature spectrum, including regions historically deemed unsuitable for pure CO_2_ systems.

The remainder of this article is structured as follows: Section 2 presents the system model, methodological framework, and thermophysical property data for the CO_2_-based mixtures. Section 3 discusses the simulation results, including performance trends under varying ambient conditions and exergy analysis of the respective mixture compositions. Finally, Section 4 summarizes the key outcomes and provides recommendations for future research on s-CO_2_ cycle optimization in low-temperature environments.

## 2. Methodology

### 2.1. Cycle Configuration

**Figure 1:**
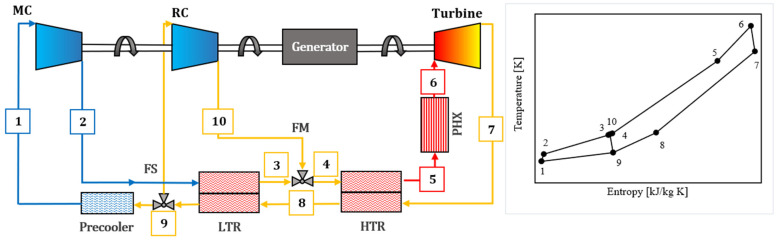
Recompression Brayton power cycle and T-s diagram [37].

A recompression Brayton cycle (Figure 1) employing supercritical CO_2_ as the baseline working fluid was modeled [37,38]. The system configuration comprises a low-temperature (LT) recuperator, a high-temperature (HT) recuperator, a main compressor, a recompressor, a primary heat source that elevates the fluid temperature prior to turbine expansion, and a cooler that reduces the fluid temperature and pressure following expansion and recuperation, preceding compression.

Pinch points in the LT and HT recuperators were closely monitored to ensure adequate temperature differentials for effective heat transfer. The overall heat transfer conductance (UA) of each recuperator was assigned according to design constraints.

### 2.2. Recompression Brayton Cycle Simulation Methodology

The supercritical CO_2_ recompression Brayton cycle (RBC) is modeled using a steady-state, sequential modular simulation approach. In this framework, each component is solved in sequence along the working fluid’s flow path, while applying conservation laws at defined thermodynamic state points. The RBC configuration incorporates two parallel compressors: a main compressor and a recompressor, the latter processing the portion of the flow that bypasses the precooler. This split-flow arrangement establishes a recirculation loop, closed by mixing the recompressed stream with the main compressor discharge upstream of the high-temperature recuperator (HTR).

To handle this recycle loop, a tear stream—corresponding to the split flow—is defined, and an iterative solution method is employed. The simulation begins with an initial estimate of the split fraction or an intermediate state variable (e.g., recuperator outlet temperature), and then iteratively solves the cycle until convergence is achieved within a specified tolerance.

Each simulation starts with defined boundary conditions, such as the maximum turbine inlet temperature, system high and low pressures, and ambient cooler temperature. The thermodynamic states of all cycle points are initialized accordingly. The solver progresses sequentially through the components. For instance, given the inlet conditions, turbine expansion is computed first to obtain the outlet state. This is followed by compression of the main compressor intake (from the precooler outlet) up to the high pressure, yielding the discharge state. Using these outputs, the HTR is solved based on the initial guess of the recompression loop variable.

Subsequently, the model evaluates the recompressor outlet—compressing the bypassed flow—and computes the performance of the low-temperature recuperator (LTR). The two resulting streams—recompressor outlet and LTR outlet—are mixed at equal pressure to form a single stream entering the cold side of the HTR. The iterative loop then adjusts the guessed variable (e.g., recompression mass fraction or LTR outlet temperature) and resolves the components until energy balance in both recuperators and mass consistency across branches are satisfied.

This iterative process ensures full convergence of the recompression loop and compliance with all design targets, such as matching outlet temperatures or heat duties within defined residuals. The outcome is a complete set of converged thermodynamic properties throughout the cycle that satisfy all component and loop closure equations.

### 2.3. Component Modeling

The cycle model includes all principal components of a recompression Brayton cycle: a turbine, main compressor, recompressor, two recuperative heat exchangers (high- and low-temperature recuperators), a primary heat exchanger (heat source), and a precooler (heat sink). Each component is represented by steady-state thermodynamic relationships that characterize its performance and associated constraints.

The **turbomachinery models** (turbine and compressors) are modeled using isentropic efficiency definitions in conjunction with specified pressure ratios. For the turbine, expansion is assumed adiabatic, and the actual outlet enthalpy is determined from the isentropic enthalpy change scaled by the efficiency. This yields the specific work output and exit temperature. Similarly, the main compressor and recompressor employ isentropic relationships to calculate the required work input and outlet states based on the pressure rise and efficiency. These calculations are enthalpy-based and ensure that each device operates according to prescribed performance parameters. Mass continuity is enforced such that the total turbine mass flow equals the sum of the flows through the two compressors, and the flow division between them is consistent with the resolved split fraction.

Heat exchangers are simulated via energy balances and heat transfer correlations. The **recuperators (HTR and LTR)** transfer heat between the hot exhaust stream and the cooler compressed stream of the working fluid. Each recuperator is characterized by an overall heat conductance (UA value) or an effectiveness value (a measure of heat exchanger performance). A UA-based formulation is used here, enforcing that the heat released by the hot stream equals the heat absorbed by the cold stream, under the assumption of no heat loss to the environment due to well-insulated units. Due to the temperature dependence of the fluid’s specific heat, outlet temperatures cannot be derived analytically and must instead be computed iteratively by solving the coupled energy balance and UA relationship. The model adjusts the outlet temperature guess iteratively until the computed heat transfer matches the target UA within a strict numerical tolerance. In practice, this is done by computing the heat transfer for a trial outlet temperature, evaluating the imbalance or residual (e.g., the difference between required and achieved UA or the energy imbalance), and then using a root-finding method to correct the estimate. A combination of bisection (bracketing) and secant iterations is employed to ensure robust and efficient convergence of this non-linear problem. If the specified UA of a recuperator is effectively zero (below a threshold, e.g., $1\times 10^{-12}$), the component is treated as inactive (bypassed) in the model—meaning no heat is exchanged in that recuperator. This allows the code to seamlessly handle configurations where one of the recuperators is omitted by simply setting its UA to a negligible value.

The **primary heat exchanger** (also referred to as the hot source) supplies thermal energy from an external source to raise the working fluid temperature to the turbine inlet set point. It is modeled through an energy balance, bringing the fluid from the HTR outlet to the desired turbine inlet state. The heat input is computed as the product of the mass flow rate and enthalpy rise. A pressure drop may also be imposed, either as a fixed value or as a fraction of inlet pressure, to simulate realistic flow resistance between the compressor discharge and turbine inlet.

The **precooler** removes waste heat from the working fluid to the ambient sink—such as cooling water or air—on the low-temperature side of the cycle. It cools the fluid from the LTR outlet to the main compressor inlet temperature. This component is modeled using an energy balance that determines the rejected heat and ensures the desired inlet conditions for compression are achieved. A pressure loss may also be assigned. Both the heater and precooler are assumed to operate under steady-state conditions with negligible environmental heat losses.

Together, these components form a complete set of algebraic equations that govern the thermodynamic behavior of the working fluid throughout the recompression Brayton cycle.

### 2.4. Performance Calculation

Once the thermodynamic state points are fully resolved, the performance of the recompression Brayton cycle is evaluated based on both component-level and system-wide metrics. The **specific work output** of the turbine is determined using an isentropic expansion model, subsequently adjusted by the turbine isentropic efficiency ($\eta_{turb}$) to account for real-world deviations from ideal behavior:

(1) $$w_{turb}=\eta_{turb}\cdot (h_{turb,in}-h_{turb,out,isentropic})$$

Similarly, the **specific work input** for both the main compressor and recompressor is evaluated using the following:

(2) $$w_{comp}=\frac{h_{comp,in}-h_{comp,out,isentropic}}{\eta_{comp}}$$

These values are multiplied by the respective mass flow rates to calculate the component-wise **power outputs and consumptions:**

(3) $${\dot{W}}_{turb}={{\dot{m}}_{turb}\cdot w}_{turb}$$

(4) $${\dot{W}}_{MC}={{\dot{m}}_{MC}\cdot w}_{MC}$$

(5) $${\dot{W}}_{RC}={{\dot{m}}_{RC}\cdot w}_{RC}$$

The **total net power output** is therefore as follows:

(6) $${\dot{W}}_{net}={\dot{W}}_{turb}-|{\dot{W}}_{MC}+{\dot{W}}_{RC}|$$

where:

(7) $${\dot{m}}_{RC}=\gamma \cdot {\dot{m}}_{turb}$$

(8) $${\dot{m}}_{MC}={\dot{m}}_{turb}-{\dot{m}}_{RC}$$

The **thermal efficiency of the cycle** is defined according to the first law of thermodynamics as the ratio of the net power output to the heat input delivered in the primary heat exchanger (PHX):

(9) $$\eta_{th,cycle}=\frac{{\dot{W}}_{net}}{{\dot{Q}}_{PHX}}$$

where the heat duty in the PHX is

(10) $${\dot{Q}}_{PHX}={\dot{m}}_{turb}\cdot (h_{PHX,out}-h_{PHX,in})$$

For the recuperative heat exchangers (HTR and LTR), the heat transfer rate is determined by the energy balance between the hot and cold streams:

(11) $${\dot{Q}}_{HX}={\dot{m}}_{hot}\cdot (h_{hot,out}-h_{hot,in})$$

The corresponding *UA* evaluation utilizes the logarithmic mean temperature difference (LMTD) method:

(12) $${\dot{Q}}_{HX}=UA\cdot \Delta T_{lm}$$

(13) $$\Delta T_{lm}=\frac{\left(T_{h,in}-T_{c,out}\right)-\left(T_{h,out}-T_{c,in}\right)}{\ln\left(\frac{T_{h,in}-T_{c,out}}{T_{h,out}-T_{c,in}}\right)}$$

where *UA* is iteratively adjusted to meet the specified performance criteria, using a combination of bisection and secant methods, with convergence achieved when the residual is below a defined tolerance:

(14) $$\frac{\left|Residual_{HX}\right|}{UA_{target}}<1\cdot 10^{-12}$$

Furthermore, the **recuperator effectiveness** ($\varepsilon_{HX}$) is computed as

(15) $$\varepsilon_{HX}=\frac{{\dot{Q}}_{actual}}{{\dot{Q}}_{max}\;}$$

(16) $${\dot{Q}}_{max}=C_{min}\cdot (T_{h,in}-T_{c,out})$$

where

(17) $$C_{min}=min({\dot{m}}_{hot}\cdot c_{p,hot},\;{\dot{m}}_{cold}\cdot c_{p,cold})$$

All component **pressure drops** are incorporated into the thermodynamic property calculations, applied either as absolute or relative drops, as follows:

(18) $$P_{out}=P_{in}-\Delta P_{abs}$$

(19) $$P_{out}=P_{in}\cdot (1-\Delta P_{abs})$$

where applicable; when a heat exchanger’s UA falls below a predefined negligible threshold ($1\cdot 10^{-12}$), the component is considered **inactive** and bypassed within the model.

Finally, in cases where a **target net power output** is prescribed, the model employs an iterative adjustment of the total mass flow rate until the calculated net power converges to the desired value within a predefined numerical tolerance. This procedure ensures consistency between the system-level performance requirement and the thermodynamic state configuration, allowing the model to scale the cycle appropriately while preserving component efficiency and design constraints:

(20) $$Residual_{power}={\dot{W}}_{net.calculated}-{\dot{W}}_{net,target}$$

This ensures that the simulated cycle performance reflects both the specified design point and the thermodynamic behavior of the working fluid under the given conditions, capturing the complex interplay of heat exchanger performance, compressor work, turbine expansion, and system irreversibilities.

The exergetic analysis was conducted in accordance with the relevant equations pertaining to the Second Law of Thermodynamics [39]. Furthermore, the assumption of a steady-state and adiabatic system was made. The exergy balance is expressed in Equation (21).

(21) $$0={\dot{E}}_{Q}-{\dot{E}}_{W}-{\dot{E}}_{D}+\sum {\dot{m}}_{in}\cdot e_{in}-\sum {\dot{m}}_{out}\cdot e_{out}$$

where ${\dot{E}}_{Q}$ denotes the exergy rate of the heat transfer, ${\dot{E}}_{W}$ is the exergy rate of the work, ${\dot{E}}_{D}$ signifies the exergy destruction rate, and e is the specific exergy. The specific exergy and exergy values at each state point (i) of the cycle are calculated by Equations (22) and (23), respectively.

(22) $$e_{i}=\left(h-h_{o}\right)-T_{o}\cdot \left(s-s_{o}\right)$$

(23) $${\dot{E}}_{i}=\dot{m}\cdot e_{i}$$

The following expressions provide a quantitative representation of the exergetic efficiency of the individual components that constitute the given cycle [40]:

(24) $$\eta_{ex,turb}=\frac{{\dot{W}}_{turb}}{{\dot{m}}_{turb}\cdot \left(e_{6}-e_{7}\right)}$$

(25) $$\eta_{ex,MC}=\frac{{\dot{m}}_{MC}\cdot \left(e_{1}-e_{2}\right)}{{\dot{W}}_{MC}}$$

(26) $$\eta_{ex,RC}=\frac{{\dot{m}}_{RC}\cdot \left(e_{9}-e_{10}\right)}{{\dot{W}}_{RC}}$$

(27) $$\eta_{ex,HTR}=\frac{\left(e_{5}-e_{4}\right)}{\left(e_{7}-e_{8}\right)}$$

(28) $$\eta_{ex,LTR}=\frac{\left(e_{3}-e_{2}\right)}{\left(e_{8}-e_{9}\right)}$$

The exergy efficiency of the cycle is defined as the ratio of thermal efficiency to the Carnot efficiency of the cycle [39,41].

(29) $$\eta_{ex,cycle}=\frac{\eta_{th,cycle}}{\eta_{Eq-Carnot}}$$

The value of $\eta_{Eq-Carnot}^{dis}$ represents the equivalent Carnot efficiency of the cycle [39,41,42,43,44]. This can be calculated using Equation (30).

(30) $$\eta_{Eq-Carnot}=1-\frac{T_{rej}^{dis}}{T_{abs}^{dis}}$$

In this Equation (30), $T_{rej}^{dis}$ denotes the heat rejection temperature and $T_{abs}^{dis}$ signifies the temperature at which heat is absorbed during the cycle in the discharge stage. The values of these temperatures are determined by means of the following equations:

(31) $$T_{rej}=\frac{\int_{8r}^{5}T\cdot ds}{s_{5}-s_{8r}}$$

(32) $$T_{abs}=\frac{\int_{5}^{6}T\cdot ds}{s_{6}-s_{5}}$$

The Second Law of Thermodynamics provides a framework for quantifying the impact of irreversibilities—such as those arising from heat transfer and fluid flow—on the overall performance of thermodynamic cycles [42]. To account for these effects, the concept of an equivalent Carnot cycle, as introduced in Equation (30), must be extended and refined as follows:

(33) $$\eta_{th}=\eta_{Eq-Carnot}-\frac{T_{rej}\cdot {\dot{\sigma }}_{total}}{{\dot{Q}}_{PHX}}$$

In order to determine the exergy destruction rate of the components of the examined cycle, the following equations are employed [40]:

(34) $${\dot{E}}_{D,turb}={\dot{m}}_{turb}\cdot \left(e_{6}-e_{7}\right)-{\dot{W}}_{turb}$$

(35) $${\dot{E}}_{D,MC}={\dot{m}}_{MC}\cdot \left(e_{1}-e_{2}\right)-{\dot{W}}_{MC}$$

(36) $${\dot{E}}_{D,RC}={\dot{m}}_{MC}\cdot \left(e_{9}-e_{10}\right)-{\dot{W}}_{RC}$$

(37) $${\dot{E}}_{D,HTR}={\dot{m}}_{turb}\cdot \left(\left(e_{4}-e_{5}\right)+\left(e_{7}-e_{8}\right)\right)$$

(38) $${\dot{E}}_{D,LTR}={\dot{m}}_{turb}\cdot \left(e_{2}-e_{3}\right)+{\dot{m}}_{MC}\cdot \left(e_{8}-e_{9}\right)$$

### 2.5. Fluid Blends Properties

The performance of the supercritical CO_2_ Brayton cycle is highly sensitive to the thermophysical properties of the working fluid, especially in the vicinity of its critical point. This section presents a comparative analysis of four CO_2_-based binary mixtures—CO_2_/Kr, CO_2_/CF_4_, CO_2_/CH_4_, and CO_2_/NF_3_—selected for their capacity to modify critical properties and enhance cycle performance under cold ambient conditions or in high-efficiency operating regimes. Thermophysical properties of all mixtures were calculated using REFPROP v10. Polynomial regressions were applied to both experimental and simulation-derived data to identify property trends as a function of molar composition. Table 1 summarizes the critical temperature, pressure, and density of the pure components utilized in the binary mixtures.

**Figure 2:**
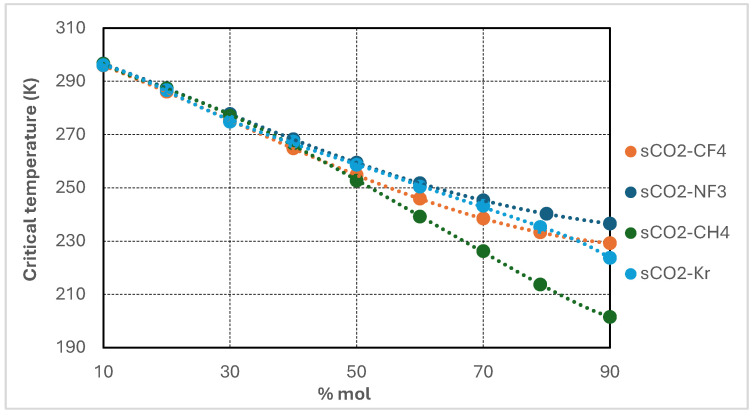
Critical temperature vs. blend chemical composition (% molar).

**Figure 3:**
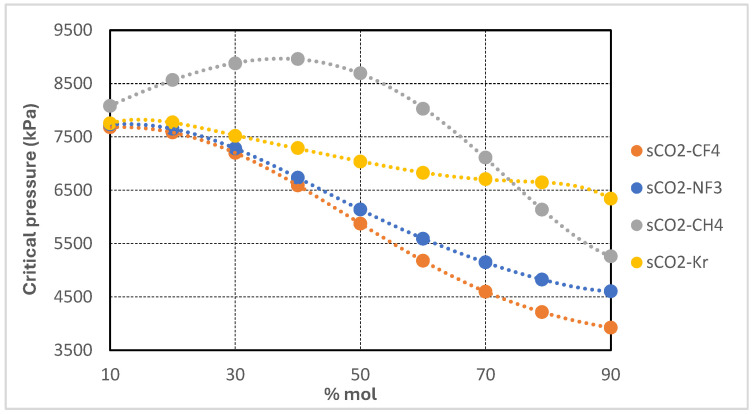
Critical pressure vs. blend chemical composition (% molar).

**Figure 4:**
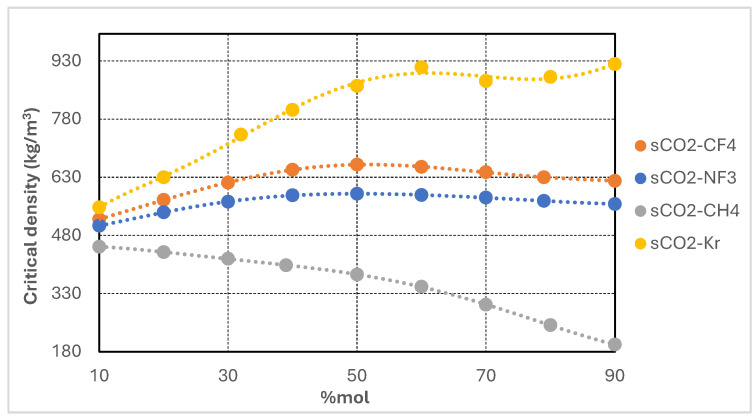
Critical density vs. blend chemical composition (% molar).

**Figure 5:**
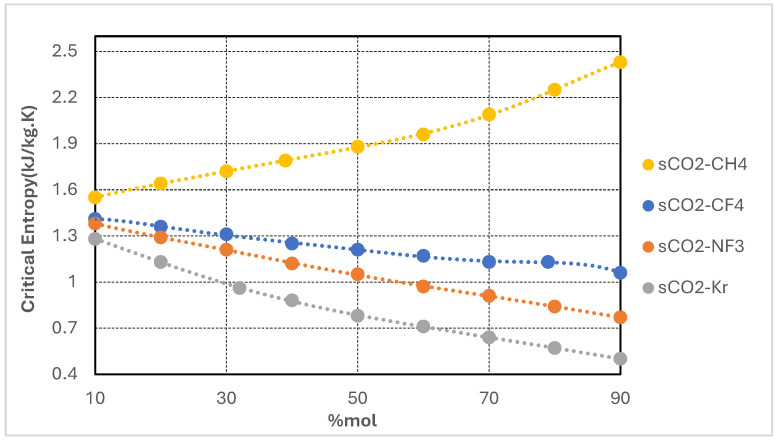
Critical entropy vs. blend chemical composition (% molar).

Each additive exhibits a significantly lower critical temperature than pure CO_2_, while the corresponding critical pressures and densities vary according to molecular structure and polarity. The objective of binary mixing is to depress the pseudo-critical temperature of the resulting mixture, thereby improving compatibility with cold heat sink conditions without substantially increasing compression power requirements. The variation of critical properties with respect to the additive molar fraction was systematically modeled and is presented in Figure 2, Figure 3, Figure 4 and Figure 5. For each CO_2_-based binary mixture, the critical temperature, pressure, and density exhibit nonlinear trends as a function of composition, reflecting the complex thermodynamic interactions between the base fluid and the additive species.

Figure 2 illustrates the variation in critical temperature of supercritical CO_2_-based binary mixtures as a function of additive molar concentration, ranging from 10 to 90 mol%. All four investigated mixtures—CO_2_/CH_4_, CO_2_/CF_4_, CO_2_/NF_3_, and CO_2_/Kr—exhibit a consistent decline in critical temperature with increasing additive content. This trend reflects the dilution effect imparted by the lower-critical-temperature additives on the CO_2_-rich base fluid.

Among the mixtures, CO_2_/CH_4_ displays the most pronounced reduction, with the critical temperature decreasing to approximately 205 K at 90 mol% CH_4_. CO_2_/CF_4_ and CO_2_/NF_3_ mixtures also demonstrate substantial depressions, reaching values in the range of 220–230 K at high additive concentrations. By contrast, the CO_2_/Kr mixture shows the most moderate response, maintaining critical temperatures above 240 K even at 90 mol% Kr—consistent with krypton’s relatively higher critical temperature.

These results confirm that incorporating high proportions of low-critical-temperature additives is an effective strategy for lowering the pseudo-critical point of the working fluid. This enables stable supercritical operation under colder ambient conditions and broadens the operational envelope of recompression Brayton cycles in subzero environments.

Figure 3 presents the variation in critical pressure of supercritical CO_2_-based binary mixtures as a function of additive molar concentration. The four studied mixtures—CO_2_/CH_4_, CO_2_/CF_4_, CO_2_/NF_3_, and CO_2_/Kr—exhibit distinct trends with increasing additive content. In the case of the CO_2_/CH_4_ mixture, the critical pressure initially rises slightly, peaking at approximately 40 mol% CH_4_, before decreasing significantly at higher concentrations. This non-monotonic behavior is characteristic of systems where the additive possesses a substantially lower critical pressure than CO_2_ and engages in complex phase interactions.

Conversely, the CO_2_/CF_4_ and CO_2_/NF_3_ mixtures display a more regular and nearly linear decline in critical pressure, with CO_2_/CF_4_ exhibiting the steepest reduction—falling below 4000 kPa at 90 mol% concentration. The CO_2_/Kr mixture shows the mildest response, maintaining critical pressures above 6000 kPa across the full composition range, consistent with krypton’s relatively higher critical pressure.

These trends hold important implications for turbomachinery design in recompression Brayton cycles. Mixtures with depressed critical pressures, such as CO_2_/CF_4_ and CO_2_/NF_3_ at high additive concentrations, allow for reduced compressor inlet pressures, thereby lowering specific compression work and enabling the use of smaller pressure ratios. This reduction in mechanical demand can translate into decreased component stress and more compact compressor architectures. However, lower operating pressures also result in reduced fluid density—particularly at the compressor inlet—which may increase volumetric flow rates and require larger turbomachinery to accommodate the same mass flow.

In contrast, mixtures like CO_2_/Kr, which retain higher critical pressures, necessitate elevated inlet pressures and pressure ratios, leading to increased compression work and more demanding mechanical designs. The non-monotonic behavior of the CO_2_/CH_4_ mixture warrants careful analysis during component sizing and system design, as the optimal pressure levels shift with mixture composition. Overall, the ability to modulate the cycle’s critical pressure through binary composition offers valuable flexibility in turbomachinery optimization, facilitating trade-offs among pressure ratio, specific work, volumetric flow capacity, and mechanical integrity.

Figure 4 illustrates the variation in critical density of supercritical CO_2_-based binary mixtures as a function of additive molar concentration. The four analyzed mixtures—CO_2_/CH_4_, CO_2_/CF_4_, CO_2_/NF_3_, and CO_2_/Kr—exhibit markedly different trends with increasing additive content. The CO_2_/Kr mixture demonstrates the highest increase in critical density, exceeding 880 kg/m^3^ at high krypton concentrations, attributable to krypton’s intrinsically high critical density. This behavior is advantageous for cycle components, as increased working fluid density enhances compressor suction conditions, reduces volumetric flow rates, and facilitates more compact turbomachinery and heat exchanger designs. These benefits support more efficient compression stages and a reduced equipment footprint.

In contrast, the CO_2_/CH_4_ mixture exhibits a continuous decline in critical density with increasing CH_4_ content, dropping below 200 kg/m^3^ at 90 mol% CH_4_. This substantial reduction presents engineering challenges, including elevated volumetric flow rates, larger compressor sizes, and higher flow velocities, which can increase pressure losses in piping and heat exchangers. Such effects may partially offset thermodynamic gains obtained from lower critical temperatures and pressures by introducing greater irreversibilities and larger component dimensions.

The CO_2_/CF_4_ and CO_2_/NF_3_ mixtures display intermediate behavior, with moderate increases in critical density at low to mid-range additive contents (peaking near 50 mol%), followed by slight decreases at higher concentrations. These results suggest that favorable density values can be achieved at moderate blend ratios, offering a balance between reduced compression work and compact component design.

Overall, critical density trends play a pivotal role in determining power cycle layout, turbomachinery design, and system efficiency. Mixtures yielding higher critical densities—such as CO_2_/Kr and CO_2_/NF_3_ at moderate concentrations—support improved volumetric efficiency, reduced pumping requirements, and lower pressure drop penalties, facilitating more compact and efficient cycle configurations. Conversely, mixtures with markedly reduced critical densities—most notably CH_4_-rich blends—require careful consideration to mitigate design penalties, despite their potential to enhance thermodynamic performance under extremely low ambient temperatures.

Figure 5 depicts the variation in critical entropy of supercritical CO_2_-based binary mixtures as a function of additive molar concentration for the CO_2_/CH_4_, CO_2_/CF_4_, CO_2_/NF_3_, and CO_2_/Kr systems. Two distinct trends are observed. The CO_2_/CH_4_ mixture exhibits a monotonic increase in critical entropy, reaching values exceeding 2.4 kJ/kg·K at high CH_4_ concentrations. This behavior reflects the broadening of the two-phase envelope and the increased molecular disorder near the critical point, characteristic of light, highly volatile additives such as methane. In contrast, the CO_2_/Kr, CO_2_/NF_3_, and CO_2_/CF_4_ mixtures demonstrate a progressive decline in critical entropy with increasing additive content, with the CO_2_/Kr blend exhibiting the lowest entropy values across the entire composition range.

From a thermodynamic standpoint, critical entropy serves as an indicator of fluid expansivity and compressibility in the vicinity of the critical point. Mixtures with higher critical entropy—such as CO_2_/CH_4_—tend to undergo greater specific volume changes during expansion, potentially increasing turbine enthalpy drops and enhancing work output. However, this also leads to larger volumetric flow rates, posing challenges for turbine design and potentially reducing mechanical efficiency if not properly mitigated. Conversely, mixtures with lower critical entropy—such as CO_2_/Kr and CO_2_/NF_3_—exhibit more compact expansion behavior, characterized by lower expansion ratios and reduced entropy generation. This is advantageous for narrower turbine blade designs, minimizes irreversibilities in expansion processes, and supports more precise control under fluctuating loads or ambient conditions. Lower entropy is also associated with steeper isentropic slopes, enhancing cycle stability and regulation.

In summary, variations in critical entropy provide valuable insights into the influence of fluid composition on expansion dynamics, entropy production, and exergy performance. Thus, the selection of an optimal working fluid blend must account not only for thermodynamic advantages derived from lower critical temperatures, but also for entropy-related implications on turbomachinery design, recuperator integration, and overall cycle controllability.

### 2.6. Thermodynamic Modeling Validation

**Figure 6:**
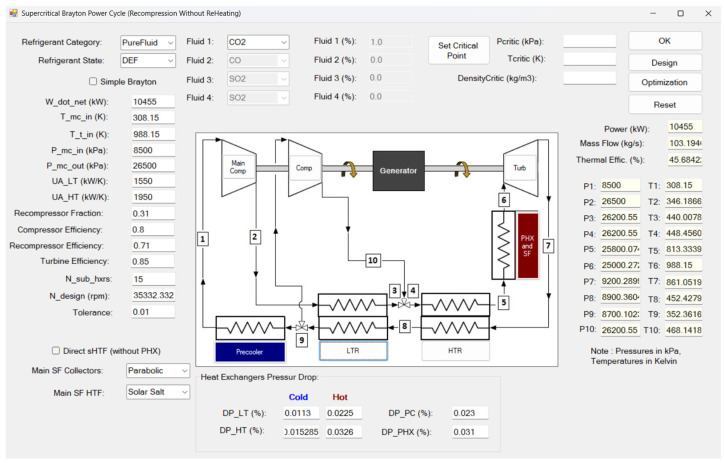
SCSP software [38]. Thermodynamic modelling verification and validation based on experimental data provided in the 10 MWe STEP pilot plant (Sandia Test Facility) [46].

A recompression s-CO_2_ Brayton cycle was thermodynamically modeled to evaluate performance under cold ambient conditions [37,38]. The simulation interface, implemented using SCSP software version 2.0, is shown in Figure 6. The cycle configuration consists of a main compressor, a recompressor, a single turbine, two recuperative heat exchangers—a low-temperature recuperator (LTR) and a high-temperature recuperator (HTR)—and a precooler (gas cooler), arranged in a closed-loop layout. High- and low-side pressures were fixed according to design specifications, while the flow split to the recompressor was optimized to maximize net cycle efficiency.

In the steady-state framework, pressures, temperatures, and mass flow rates at all major state points—including compressor inlets/outlets, turbine inlet/outlet, and recuperator interfaces—were determined by applying component-wise energy balances and isentropic relations. To ensure accurate evaluation of thermophysical properties across the relevant pressure and temperature ranges, the NIST REFPROP v10 (Reference Fluid Thermodynamic and Transport Properties) database was integrated into the model [45]. REFPROP v10 supplies high-fidelity equations of state for both pure CO_2_ and mixtures, delivering precise values for density, enthalpy, entropy, specific heat capacities, and other key properties.

By incorporating REFPROP’s real-fluid property routines, the model accounts for non-ideal gas behavior, particularly near the critical region and in the presence of additive gases. This is essential for capturing phenomena such as abrupt property variations at low temperatures and high pressures, which critically influence s-CO_2_ cycle performance. The use of REFPROP is especially important for CO_2_-based fluid mixtures, as it enables rigorous computation of mixture-dependent properties—such as pseudocritical temperature and variable heat capacities—using validated mixture models. This integration significantly enhances the realism and predictive accuracy of the cycle simulations.

**Figure 7:**
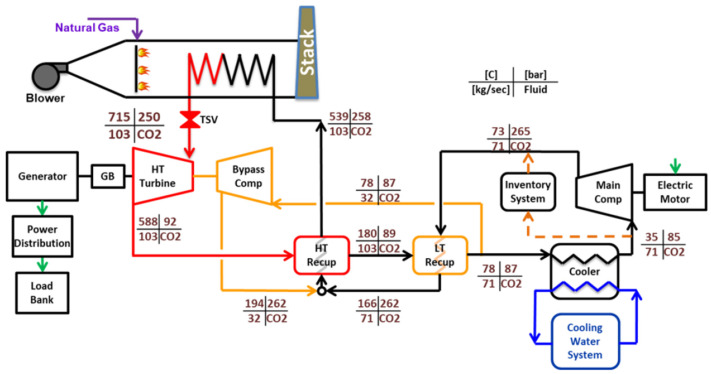
Experimental data from the 10 MWe STEP pilot plant (Sandia Test Facility) commissioned in 2024 [46].

Model validation was performed through comparison with experimental data obtained from the 10 MWe STEP pilot plant at the Sandia Test Facility (Figure 7), commissioned in 2024 [46]. This indirect-fired s-CO_2_ Brayton facility—the largest of its kind—provided temperature measurements at key thermodynamic state points corresponding to a recompression cycle configuration. The predicted state temperatures from the SCSP simulation exhibited excellent agreement with the STEP pilot data, with deviations typically within ±1.2 K of the measured values.

This close correspondence between model predictions and experimental results lends strong confidence to the accuracy of the implemented thermodynamic formulations and property evaluations based on REFPROP’s real-gas modeling. The validated model thus constitutes a robust tool for analyzing cycle performance enhancements and identifying potential operational challenges arising from modifications to the working fluid composition or operating conditions, particularly in cold-climate scenarios.

As shown in Table 2, a comparative analysis is conducted between the results obtained using the SCSP software and those reported by the Sandia Test Facility, which serves as the experimental reference [46]. The objective of this comparison is to quantify the relative discrepancies between the two data sets and evaluate the accuracy of the simulation framework.

### 2.7. Operating Conditions

The simulations were configured to replicate the operating conditions of the 10 MWe STEP pilot plant at Sandia National Laboratories [46], thereby ensuring consistency and comparability with established experimental benchmarks. Accordingly, the recompression Brayton cycle simulations were carried out under the conditions outlined in Table 3. The target net electrical power output was set at 10,455 kW. Accounting for a generator efficiency of 96%, this corresponds to a delivered net electrical output of 10,036.8 kW to the grid.

The turbine inlet temperature (TIT) was specified at 988.15 K (715 °C), representing typical high-temperature heat sources applicable to concentrated solar power and advanced nuclear systems. The outlet pressure of the main compressor ($P_{MC,out}$) was fixed at 26.5 MPa, while the compressor inlet pressure (CIP) was defined as 2 bar above the critical pressure of the working fluid mixture. This condition ensured that the entire cycle remained fully within the supercritical regime, thereby eliminating the risk of phase instability associated with subcritical operation.

The recuperator conductance (UA) values were initially assigned based on data from the STEP pilot plant [46], with the low-temperature recuperator ($UA_{LT}$) set at 1550 kW/K and the high-temperature recuperator ($UA_{HT}$) at 1950 kW/K. This allocation reflects a deliberate emphasis on maximizing heat recovery at higher temperature differentials via the HTR. Additionally, parametric variations of the UA values were performed to evaluate the sensitivity of cycle efficiency to recuperator performance, while consistently enforcing compliance with pinch-point constraints during the analysis.

Isentropic efficiencies for the turbomachinery components were specified according to benchmark values for s-CO_2_ Brayton systems operating under similar conditions: 71% for the main compressor, 80% for the recompressor, and 85% for the turbine. These values account for the performance degradation due to real-gas effects near the critical point, as well as practical limitations associated with component scaling and manufacturing tolerances.

The recompression fraction was optimized across simulations to determine the most effective flow split. This parameter is critical for improving recuperator effectiveness, as it directs a portion of the working fluid to bypass the precooler, thereby reducing exergy destruction associated with low-temperature heat rejection.

Pressure losses in all heat exchangers were modeled as relative drops, in line with conventional design methodologies. For the LTR, the cold-side and hot-side pressure drops were set at 1.13% and 2.25%, respectively, relative to their inlet pressures. The HTR imposed cold- and hot-side pressure drops of 1.5285% and 3.26%, respectively. The precooler and primary heat exchanger (PHX) were assigned pressure losses of 2.3% and 3.1%, respectively. These values reflect expected flow resistance and frictional effects, which are particularly relevant in high-density flow conditions characteristic of supercritical cycles.

Collectively, these simulation parameters establish a realistic and comprehensive framework for assessing the thermodynamic performance and design considerations of the recompression Brayton cycle with CO_2_-based mixtures, capturing both the thermophysical and mechanical constraints typical of large-scale power systems.

### 2.8. Performance Metrics

The thermodynamic performance of each cycle configuration was assessed using a set of key performance metrics:

**CIT** and **CIP**: These parameters critically influence the thermodynamic state of the working fluid with respect to its critical point. CIT governs fluid density and specific heat at the compressor inlet, while CIP affects the overall pressure ratio and phase stability. Together, they determine compressor behavior, flow stability, and overall cycle operability, particularly in near-critical or subcritical regimes.

**Recompression Fraction**: This dimensionless parameter defines the proportion of total mass flow that bypasses the low-temperature recuperator and is directed through the recompressor. It is optimized to enhance the effectiveness of heat recuperation and minimize thermal irreversibilities, playing a pivotal role in improving low-temperature heat recovery.

**UA** and **Pinch Point Temperature Difference**: The UA values of the high-temperature recuperator (HTR) and low-temperature recuperator (LTR) represent their thermal conductance and serve as constraints on heat transfer capability. Pinch point temperature differences denote the minimum approach temperature between the hot and cold streams in each exchanger. Smaller pinch points indicate high recuperation effectiveness but may necessitate larger or more advanced heat exchanger designs to avoid thermal bottlenecks.

The performance of the HTR, in particular, exerts a strong influence on overall cycle efficiency. As demonstrated by Stamatellos et al. [49], optimizing HTR performance by varying UA rather than fixing the minimum temperature difference enables more efficient cycle configurations and improved pinch point control. Further investigations by Stamatellos et al. [50] underscore the importance of adopting realistic UA values to prevent efficiency losses associated with pinch point relocation, especially within the high-temperature zone of the HTR.

Collectively, these metrics characterize thermodynamic efficiency, component-level behavior, and key trade-offs in recompression Brayton cycle design. They provide a basis for comparing the relative performance of alternative working fluid mixtures under cold ambient conditions

## 3. Results and Discussion

### 3.1. Impact of CO_2_-Based Binary Mixtures on RBC Performance Under Cold Ambient Conditions

The incorporation of low-critical-temperature additives into the CO_2_ recompression Brayton cycle significantly reshapes the thermodynamic characteristics of the system, particularly under cold ambient conditions where pure CO_2_ cycles experience pronounced performance limitations due to proximity to, or transgression of, the fluid’s critical point.

The simulation results presented herein reveal the nuanced effects of such binary mixtures on overall cycle behavior, including trends in thermal efficiency, evolution of pinch points within the recuperators, variations in critical pressure and density, and their corresponding implications for turbomachinery and heat exchanger design.

This section provides a detailed analysis of the numerical outcomes, systematically comparing the four investigated CO_2_-based binary mixtures—CF_4_, CH_4_, NF_3_, and Kr—across the defined operating envelope, with particular focus on the influence of their thermophysical properties and the resulting impact on cycle performance.

#### 3.1.1. Enhancement of Cycle Thermal Efficiency with CO_2_/CF_4_ Mixture

**Figure 8:**
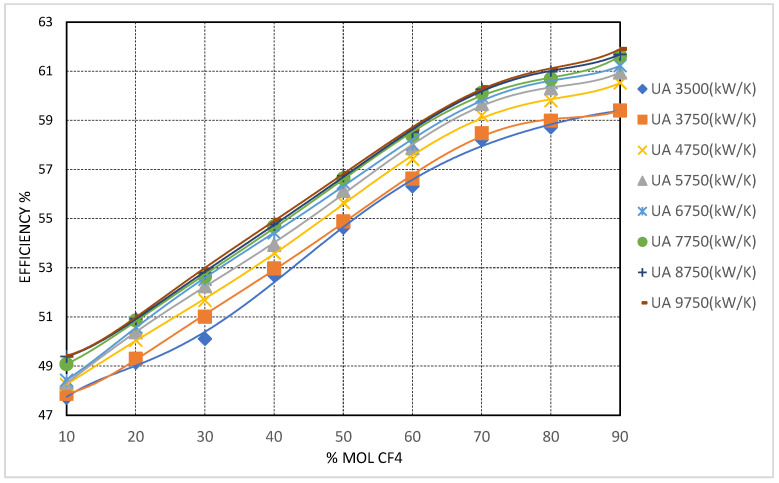
Recompression Brayton cycle thermal efficiency as a function of CF_4_ molar concentration for varying total recuperator conductance ($UA_{Total}$). Efficiency increases significantly with CF_4_ content, particularly at higher UA levels, reaching maximum values at ~80–90 mol% CF_4_. Efficiency improvements are more sensitive to UA at high additive concentrations due to increased recuperation demands at the low-temperature end.

Among the additives evaluated, tetrafluoromethane (CF_4_) demonstrates the strongest potential for enhancing the thermal efficiency of the recompression Brayton cycle under cold ambient conditions. As shown in Figure 8, increasing the molar concentration of CF_4_ in the working fluid leads to a clear and monotonic improvement in cycle efficiency. This enhancement is particularly significant at elevated recuperator conductance levels, notably when the total UA is set to 9750 kW/K, indicating that the effectiveness of CF_4_ as a working fluid additive is further amplified under well-recovered thermal configurations.

The observed enhancement in thermal efficiency resulting from the addition of tetrafluoromethane (CF_4_) is directly attributable to the significant reduction in the mixture’s critical temperature, given CF_4_’s intrinsic critical point of −42.4 °C. As the CF_4_ concentration surpasses 50 mol%, the critical temperature of the mixture drops below −20 °C, effectively eliminating the risk of subcritical operation even at extremely low compressor inlet temperatures (CIT). This behavior enables stable operation entirely within the supercritical regime at CITs as low as −40 °C, thereby preserving the favorable properties of a dense supercritical fluid while mitigating performance degradation due to two-phase flow, elevated specific work, and compressor surge risk.

At a CIT of −42.4 °C and 90 mol% CF_4_, the cycle achieves a peak thermal efficiency of approximately 60.53%, representing an improvement of nearly 14 percentage points compared to the baseline pure CO_2_ cycle under equivalent low-CIT conditions. These results underscore the viability of CF_4_-rich mixtures for high-efficiency operation in subarctic and polar environments, where ambient temperatures routinely fall below the triple point of CO_2_.

The performance of the high-temperature recuperator (HTR) in these conditions also exhibits notable improvement. The inclusion of CF_4_ moderates the temperature gradient across the HTR. This reduces the occurrence of sharp pinch points that typically limit heat transfer in pure CO_2_ systems under low-CIT conditions. However, to fully exploit these thermodynamic benefits, a substantial increase in HTR conductance is required. The results indicate that at high CF_4_ concentrations and CITs approaching −40 °C, the HTR pinch point remains a limiting factor unless the total conductance ($UA_{Total}$) exceeds 4750 kW/K.

From a turbomachinery standpoint, the reduction in mixture critical pressure—from approximately 4.74 MPa at 70 mol% CF_4_ to roughly 3.95 MPa at pure CF_4_—enables lower compressor inlet pressures (CIP), which in turn reduce the specific compression work and increase the turbine expansion ratio. This translates into higher net cycle efficiency and added flexibility in compressor design. Nonetheless, the associated increase in fluid density under these conditions requires careful consideration of volumetric flow rates and imposes mechanical design challenges, particularly in maintaining effective sealing and achieving desired compression ratios at low pressures and temperatures.

#### 3.1.2. Superior Efficiency Gains with CO_2_/CH_4_ Mixture and Challenges in Turbomachinery Integration

**Figure 9:**
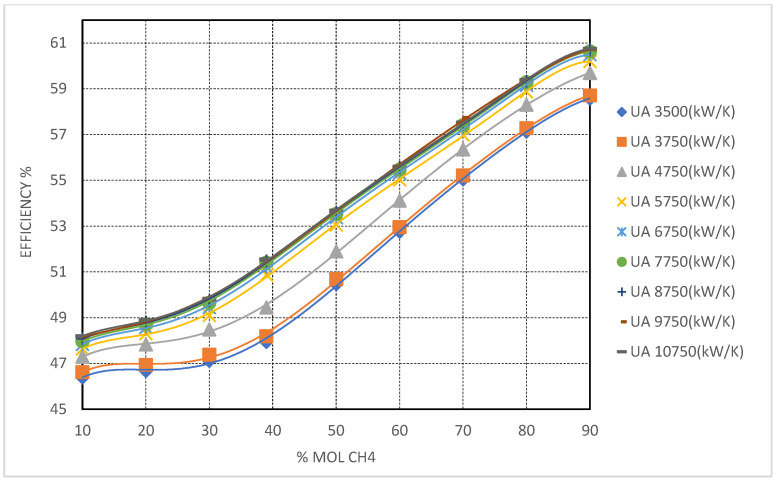
Recompression Brayton cycle thermal efficiency as a function of CH_4_ molar concentration for varying $UA_{Total}$. CH_4_-rich mixtures exhibit the highest cycle efficiency improvements among the studied blends, with efficiencies around 60% at 90 mol% CH_4_ and high UA. The efficiency sensitivity to UA is most pronounced at high CH_4_ fractions and low CIT conditions, highlighting the need for increased HTR conductance.

Methane (CH_4_) emerges as the most effective additive among the studied candidates with respect to maximizing cycle thermal efficiency. Simulation results (Figure 9) show that CO_2_/CH_4_ mixtures consistently outperform the other blends, reaching thermal efficiencies exceeding 58% at 90 mol% CH_4_ under high $UA_{total}$ conditions. These results highlight the high thermodynamic potential of CH_4_-rich mixtures; however, as will be discussed, their integration into practical turbomachinery systems introduces significant engineering challenges.

The high performance of CO_2_/CH_4_ mixtures is primarily attributed to methane’s low critical temperature (−82.6 °C or 190.6 K), which significantly reduces the mixture’s critical point even at moderate concentrations. This ensures fully supercritical operation across a broad range of cold compressor inlet temperatures (CIT), thereby avoiding two-phase flow regimes and their associated efficiency penalties. Compared to CF_4_, the efficiency gains with CH_4_ are notably more pronounced, due to the combined benefits of lower CIT and expanded turbine pressure ratios, supported by decreasing mixture critical pressures—approaching 7.3 MPa at 70 mol% CH_4_ and falling below 5 MPa at higher concentrations.

The enhanced cycle efficiency associated with CH_4_ mixtures is strongly dependent on recuperator performance. Simulation results indicate that substantial efficiency improvements are only realized when the total heat exchanger conductance ($UA_{Total}$) exceeds 5750 kW/K. This is due to the pronounced temperature glide across the high-temperature recuperator (HTR) under very low CIT conditions, which imposes significant thermal demands. At lower $UA_{Total}$ levels, the HTR pinch point becomes the dominant limiting factor, constraining heat recovery and diminishing the potential efficiency gains of the mixture.

Despite their thermodynamic advantages, CH_4_-rich mixtures pose considerable turbomachinery design challenges. The low critical pressure and, more critically, the significantly reduced fluid densities at cold operating conditions (relative to CF_4_ or NF_3_ mixtures) result in large volumetric flow rates. The simulations reveal that, at high CH_4_ concentrations and low CIT, compressor volumetric flow may increase by up to 50% compared to baseline pure CO_2_ cycles. This necessitates either larger impeller diameters, expanded flow cross-sections, or multi-stage compression strategies to preserve acceptable performance and pressure ratios. Similar considerations apply to turbine design, where larger flow passages are required, potentially lowering stage efficiency due to tip leakage and heightened sensitivity to off-design operation.

From a heat exchanger standpoint, although the more gradual temperature glide associated with CH_4_ mixtures mitigates some of the extreme pinch point constraints observed in pure CO_2_ systems, the data nonetheless indicate that over-sizing of the HTR—and to a lesser extent, the low-temperature recuperator (LTR)—is necessary to fully exploit the mixture’s thermodynamic potential. This design requirement must be carefully balanced against capital cost considerations, as oversized recuperators may offset the gains in thermal efficiency if not properly optimized.

In summary, while CH_4_-based mixtures exhibit the highest thermodynamic potential for cold-climate Brayton cycle applications, their practical integration requires significant adaptations in both turbomachinery and heat exchanger design to accommodate the altered fluid properties and flow characteristics intrinsic to methane-rich working fluids

### 3.2. Cycle Optimization Through Critical Pressure Management and Turbomachinery Implications

Expanding upon the previous analysis, critical pressure management emerged as a key parameter in optimizing the performance of the recompression Brayton cycle using CO_2_-based binary mixtures—particularly under subzero environmental conditions. The combined effect of reduced critical pressures and enhanced flexibility from lower critical temperatures led to a substantial redefinition of the compressor inlet operating window and associated turbomachinery sizing criteria. These thermodynamic trends, particularly evident in CF_4_ and CH_4_ mixtures, are quantitatively supported by Figure 8 and Figure 9, which not only display thermal efficiency improvements but also illustrate the indirect influence on turbomachinery operating ranges. Specifically, higher efficiencies were correlated with more favorable turbine expansion ratios and lower specific compression work at reduced pressures.

Among the evaluated blends, CF_4_-rich mixtures exhibited the most substantial critical pressure reduction, with values decreasing to approximately 3.95 MPa at pure CF_4_ composition. This allowed for significant compressor work savings, as the inlet pressure could be lowered below the levels permissible with pure CO_2_—without incurring the risk of subcritical operation. Moreover, despite the decline in fluid density relative to CO_2_, CF_4_’s inherently high molecular weight ensured that volumetric efficiency remained within acceptable limits under supercritical conditions.

In contrast, CH_4_-rich mixtures, although offering superior critical temperature suppression and the highest thermal efficiency gains, produced more moderate reductions in critical pressure—down to approximately 5.4 MPa at 90 mol% CH_4_. This was accompanied by a marked decrease in fluid density due to methane’s highly volatile nature, even in supercritical states. As a result, compressor volumetric flow rates increased significantly, necessitating considerable redesign of compression stages—including larger impellers and enhanced volumetric flow management strategies. These findings underscore the trade-off between thermodynamic performance and mechanical feasibility.

NF_3_ and Kr mixtures exhibited intermediate behavior. Pure NF_3_ yielded a critical pressure of approximately 4.6 MPa and offered measurable reductions in compressor work. Crucially, the high density of NF_3_ in the supercritical regime ensured that required compressor volumetric capacities remained comparable to those of pure CO_2_, thus simplifying integration. Krypton-based mixtures, while presenting the least reduction in critical pressure (remaining above 5.67 MPa at 100% Kr), provided notable advantages in terms of flow compactness. Owing to Kr’s high molar mass and density, compressor inlet volumetric flow remained low, enhancing compatibility with existing CO_2_-focused turbomachinery designs. However, the modest reductions in critical temperature and pressure translated into limited efficiency gains, as further discussed in subsequent comparative sections.

From an integrated design perspective, CF_4_-rich mixtures emerge as the most balanced solution—offering substantial efficiency improvements alongside manageable turbomachinery adaptations. While CH_4_ blends present superior thermodynamic potential, they impose considerable mechanical challenges. NF_3_ and Kr mixtures represent viable intermediate options, well-suited for applications prioritizing system simplicity, ease of retrofitting, and acceptable efficiency enhancement under cold climate conditions

### 3.3. Recuperator Performance Optimization and Sensitivity to UA

Another critical aspect assessed across all mixtures was the performance of the recuperators—particularly the sensitivity of cycle efficiency to the total heat exchanger conductance ($UA_{Total}$), which was varied parametrically from 3500 kW/K to 10,750 kW/K. The results consistently demonstrated a strong dependence of thermal efficiency on recuperator sizing, especially under cold compressor inlet temperature (CIT) scenarios.

At lower ($UA_{Total}$) values, cycle efficiency was notably limited by pinch points within the recuperators—most critically at the cold end of the low-temperature recuperator (LTR), between state points T9 and T2. This effect was most pronounced in CH_4_- and CF_4_-rich mixtures, where extreme cold CIT conditions imposed significant thermal demands on the LTR. The substantial degree of recuperation required to preheat the working fluid prior to entering the main compressor was constrained by the limited temperature differential at this pinch point, with temperatures approaching sub-ambient values. This scenario exacerbated logarithmic mean temperature difference (LMTD) limitations and pushed the system into a regime of diminishing efficiency returns.

As ($UA_{Total}$) increased, the recuperators’ ability to accommodate these thermal constraints improved significantly. Notably, when ($UA_{Total}$) surpassed 7750 kW/K, thermal efficiency exhibited a near-linear response to further conductance increases. For example, at $UA_{Total}$ = 5750 kW/K, the CO_2_–CH_4_ mixture with 90% CH_4_ achieved efficiencies above 60%, emphasizing the critical role of heat exchanger sizing in fully realizing the thermodynamic potential of these mixtures.

However, such aggressive recuperator sizing did not yield uniformly high returns across all mixtures. For Kr-based systems, efficiency gains plateaued at much lower conductance values, with limited improvements beyond $UA_{Total}$ = 3500 kW/K. This behavior is attributed to krypton’s high specific heat and density, which reduce the required heat duty across the recuperators. Consequently, Kr-based cycles can achieve near-optimal heat recovery with relatively low UA values—offering potential cost advantages in system design where Kr is the working fluid additive of choice.

**Figure 10:**
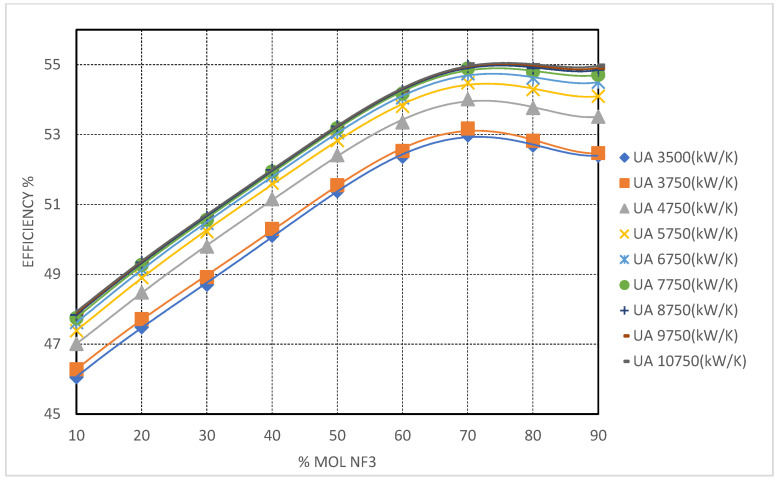
Recompression Brayton cycle thermal efficiency as a function of NF_3_ molar concentration for varying $UA_{Total}$. NF_3_-containing mixtures show robust efficiency improvements, achieving peak efficiencies around 54.5% at ~70 mol% NF_3_. While gains are lower compared to CH_4_ and CF_4_ mixtures, the trends confirm NF_3_’s effectiveness in extending the operational range of the cycle to moderate subzero CIT conditions.

NF_3_-based mixtures demonstrated intermediate behavior. Efficiency gains increased steadily up to $UA_{Total}$ ≈ 8750 kW/K, beyond which diminishing returns were observed, suggesting an optimal conductance range for these systems (see Figure 10). Furthermore, the HTR pinch point in NF_3_ blends remained more manageable than in CH_4_ and CF_4_ cases, owing to favorable thermophysical properties that facilitated more effective heat exchange near the cold end without encountering severe LMTD or pinch point penalties.

This UA sensitivity analysis further highlighted the fact that the high-temperature recuperator (HTR) dominated the cycle performance under cold CIT conditions, with the low-temperature recuperator (LTR) playing a secondary role. The required UA_HT to mitigate the cold-end pinch was significantly higher in CH_4_- and CF_4_-rich blends, often exceeding 5000 kW/K, while Kr and NF_3_ mixtures could achieve acceptable pinch management with UA_HT as low as 3500–4000 kW/K.

### 3.4. Comparative Evaluation of Mixture Performance and Operational Trade-Offs

A comprehensive comparative evaluation of the four CO_2_-based binary mixtures revealed distinct operational characteristics and trade-offs, which are critical for informing working fluid selection based on specific application requirements.

#### 3.4.1. CO_2_/CF_4_ Mixtures

CO_2_/CF_4_ mixtures emerged as the most balanced option for cold-climate applications where both thermal efficiency and mechanical feasibility are key considerations. Cycle efficiencies of up to 58% were achieved at CF_4_ concentrations between 70 and 80 mol% under high recuperator conductance conditions (see Figure 8), representing improvements of approximately 11–12 percentage points over the baseline pure CO_2_ case. Notably, CF_4_’s high fluid density resulted in compressor volumetric flow rates that, although higher than those of CO_2_, remained within the design capabilities of conventional turbomachinery.

Moreover, the favorable thermophysical properties of CF_4_—moderate critical pressure, high density, and low viscosity—confer strong compatibility with existing s-CO_2_ cycle hardware. This compatibility renders CO_2_/CF_4_ mixtures a highly attractive candidate for retrofitting existing systems for cold-environment operation, minimizing the need for extensive redesign or replacement of major components.

#### 3.4.2. CO_2_/CH_4_ Mixtures

CO_2_/CH_4_ mixtures demonstrated the most pronounced efficiency gains among all mixtures investigated, exceeding 60% at 90 mol% CH_4_ and $UA_{Total}$ values above 9750 kW/K. These high performance levels confirm CH_4_’s exceptional ability to depress the critical temperature into the cryogenic range, enabling stable supercritical operation at CITs as low as −70 °C.

However, these thermodynamic advantages come with substantial engineering challenges. The markedly low fluid density at the compressor inlet, coupled with significantly elevated volumetric flow rates, imposes severe demands on turbomachinery. Achieving the necessary compression requires larger, more complex compressor designs, as well as increased power input to maintain the target mass flow. In parallel, heat exchanger design becomes especially demanding, with CH_4_-rich mixtures requiring the highest $UA_{LT}$ values to manage sharp LTR pinch points—particularly under extreme subzero operating conditions.

As a result, while CO_2_/CH_4_ mixtures exhibit exceptional thermodynamic potential, their practical deployment is best suited to dedicated systems designed from the ground up with appropriate component scaling and performance margins. This trend is clearly evidenced in Figure 9, where the steep rise in efficiency with increasing CH_4_ concentration is accompanied by parallel increases in system complexity—highlighting the need to balance thermodynamic gains with engineering practicality.

### 3.5. Critical Temperature Reduction and Cycle Performance at Subzero Ambient Conditions

The reduction of the working fluid’s critical temperature emerges as a fundamental thermophysical parameter governing the performance of recompression Brayton cycles under cold ambient conditions. In pure CO_2_ systems, the relatively high critical temperature of 304.13 K (31.0 °C) imposes a fundamental limitation, as subzero CITs can push the working fluid into the two-phase region at the compressor inlet. This leads to severe performance degradation due to phase instability, increased compression work, and the inability to sustain the supercritical conditions required for efficient compression and recuperation.

By contrast, CO_2_-based binary mixtures incorporating additives such as CF_4_, CH_4_, NF_3_, and Kr exhibit substantial depressions in critical temperature, enabling stable supercritical operation at CITs as low as −70 °C. This capability is essential for extending the applicability of recompression Brayton cycles to cold-climate scenarios—including Arctic and Antarctic installations, high-altitude environments, or off-design winter conditions in temperate regions.

#### 3.5.1. CO_2_/CF_4_ Mixture

CO_2_/CF_4_ blends exhibit a marked decrease in critical temperature, reaching approximately 229.69 K (−43.46 °C) at pure CF_4_. Simulation results indicate that increasing CF_4_ content yields progressively smoother supercritical behavior at low CITs, avoiding abrupt phase transitions and preserving continuous heat recuperation. For instance, at 90 mol% CF_4_, the cycle attains an efficiency of approximately 57.59% under the highest tested recuperator conductance ($UA_{Total}$ = 10,750 kW/K), while maintaining minimized pinch-point temperature differences across both HTR and LTR.

This favorable performance is partly attributed to CF_4_-rich mixtures’ higher density and compressibility characteristics at low temperatures, which support acceptable compressor inlet properties even under reduced pressure. Furthermore, the substantially lowered critical pressure (∼3.95 MPa for 100% CF_4_) enables lower CIP, improving turbine expansion ratios and net cycle efficiency. However, the elevated specific heat capacity of these mixtures increases recuperation demands—particularly in the LTR—necessitating careful sizing of heat exchangers.

#### 3.5.2. CO_2_/CH_4_ Mixture

CO_2_/CH_4_ mixtures offer the greatest thermodynamic performance gains under ultra-cold conditions. With a critical temperature of 191.91 K (−81.24 °C) for pure CH_4_, these mixtures enable fully supercritical cycle operation at CITs below −70 °C. At 90 mol% CH_4_, the cycle achieves a thermal efficiency of approximately 60%, representing an improvement of over 15 percentage points relative to pure CO_2_.

Nonetheless, this impressive gain is accompanied by notable engineering challenges. CH_4_-rich mixtures suffer from significantly lower fluid density, resulting in elevated volumetric flow rates through the compressor and recuperators. Accommodating these flows demands larger turbomachinery dimensions, increased heat exchanger surface areas, and an expanded system footprint. These factors introduce cost and complexity trade-offs, particularly for large-scale systems. Despite this, the exceptional thermodynamic behavior of CH_4_-blends positions them as ideal candidates for specialized systems operating in extreme subzero conditions.

#### 3.5.3. CO_2_/NF_3_ Mixture

NF_3_-based mixtures provide a balanced compromise between critical temperature reduction and manageable fluid properties. With a critical temperature of 235.65 K (−37.5 °C) for pure NF_3_, these mixtures support supercritical operation down to CITs of approximately −30 °C. At NF_3_ concentrations of 70–80 mol%, cycle efficiencies of 54.44% were achieved under high $UA_{Total}$ conditions.

NF_3_-rich blends maintain relatively high fluid densities, mitigating the volumetric flow penalties encountered with CH_4_ and facilitating more compact turbomachinery design. Moreover, the recuperator pinch-point behavior remains well-contained, simplifying heat exchanger integration. These attributes render NF_3_ a compelling choice for cold, though not extreme, environments where thermal efficiency is valued but system simplicity and hardware reuse are also critical.

#### 3.5.4. CO_2_/Kr Mixture

Krypton-based mixtures exhibit the most conservative performance enhancements among the studied blends. The critical temperature reduction is modest—yielding stable supercritical behavior at CITs down to approximately −50 °C. At 90 mol% Kr, cycle efficiency reaches ∼54%, with only mild sensitivity to further increases in recuperator conductance.

**Figure 11:**
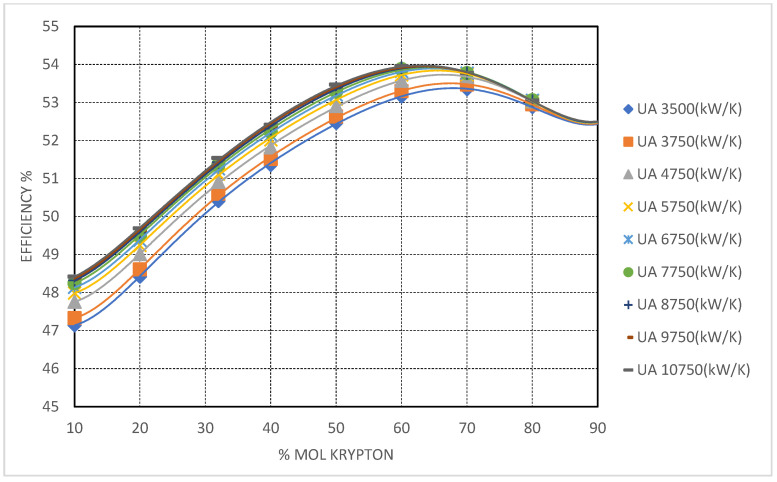
Recompression Brayton cycle thermal efficiency as a function of Kr molar concentration for varying $UA_{Total}$. CO_2_/Kr mixtures provide modest but consistent efficiency enhancements, peaking around 52–53% at 70–80 mol% Kr. Compared to other additives, the efficiency improvements are less sensitive to UA increases, reflecting Kr’s higher critical pressure and limited critical temperature reduction capacity.

The principal advantage of Kr lies in its high molar mass and density, which limit volumetric expansion at low temperatures and allow compatibility with existing s-CO_2_ turbomachinery. As evidenced in Figure 11, Kr-rich mixtures offer predictable, stable operation with minimal pinch-point challenges. However, the limited thermodynamic gains, coupled with krypton’s high cost and restricted availability, constrain its use to niche applications where specific mechanical compatibility or operational stability is prioritized over efficiency maximization.

#### 3.5.5. Comparative Performance Under Various CIT Scenarios

Analysis of the simulation results across all mixtures and compressor inlet temperature (CIT) scenarios reveals a consistent correlation between critical temperature depression and thermal efficiency enhancement. At CIT values above 0 °C, the performance differences among the mixtures are less pronounced, as all configurations operate comfortably within the supercritical region. In these conditions, pure CO_2_ cycles can still achieve acceptable efficiencies exceeding 46%.

However, as CIT decreases below −10 °C, the advantages of binary mixtures become increasingly significant. CO_2_/CH_4_ and CO_2_/CF_4_ mixtures maintain superior thermal efficiencies, while pure CO_2_ cycles experience severe performance penalties due to transitions into the two-phase region. This divergence reinforces the necessity of modifying fluid composition to preserve supercritical operation under cold ambient conditions.

Furthermore, the sensitivity of thermal efficiency to recuperator conductance ($UA_{Total}$) increases markedly at lower CITs for all mixtures. As the thermal gradient across the recuperators widens, heat exchanger performance becomes a critical constraint, with pinch-point limitations becoming more severe. These findings underscore the importance of co-optimizing working fluid composition and heat exchanger design to fully leverage the thermodynamic improvements afforded by critical temperature suppression.

### 3.6. Sensitive Exergy Analysis

This section focuses on evaluating component-level exergy destruction and exergy efficiency in the recompression Brayton cycle (RBC), providing deeper insight into the irreversibilities inherent to each subsystem.

#### 3.6.1. Pure CO_2_

**Figure 12:**
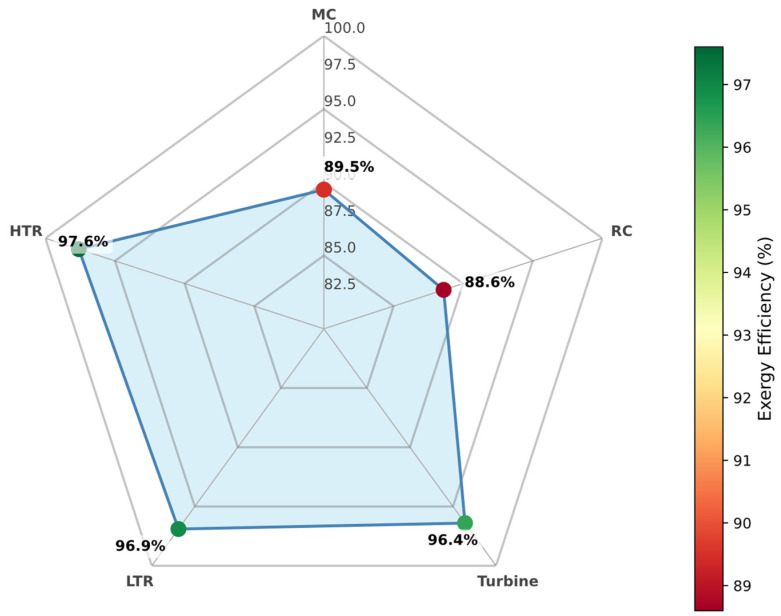
Exergy efficiency with pure s-CO_2_.

When operating with pure supercritical CO_2_ as the working fluid, the RBC configuration exhibits considerable variation in component exergy efficiencies (see Figure 12). Among the turbomachinery elements, the main compressor (MC) and recompressor (RC) demonstrate the lowest exergy efficiencies—reflecting substantial entropy generation due to compression processes near the critical point. Conversely, the high-temperature recuperator (HTR) achieves the highest exergy efficiency, underscoring the critical role of this component in recovering otherwise wasted thermal potential and minimizing irreversibilities in the high-temperature segment of the cycle.

#### 3.6.2. CO_2_/CF_4_ Mixture

**Figure 13:**
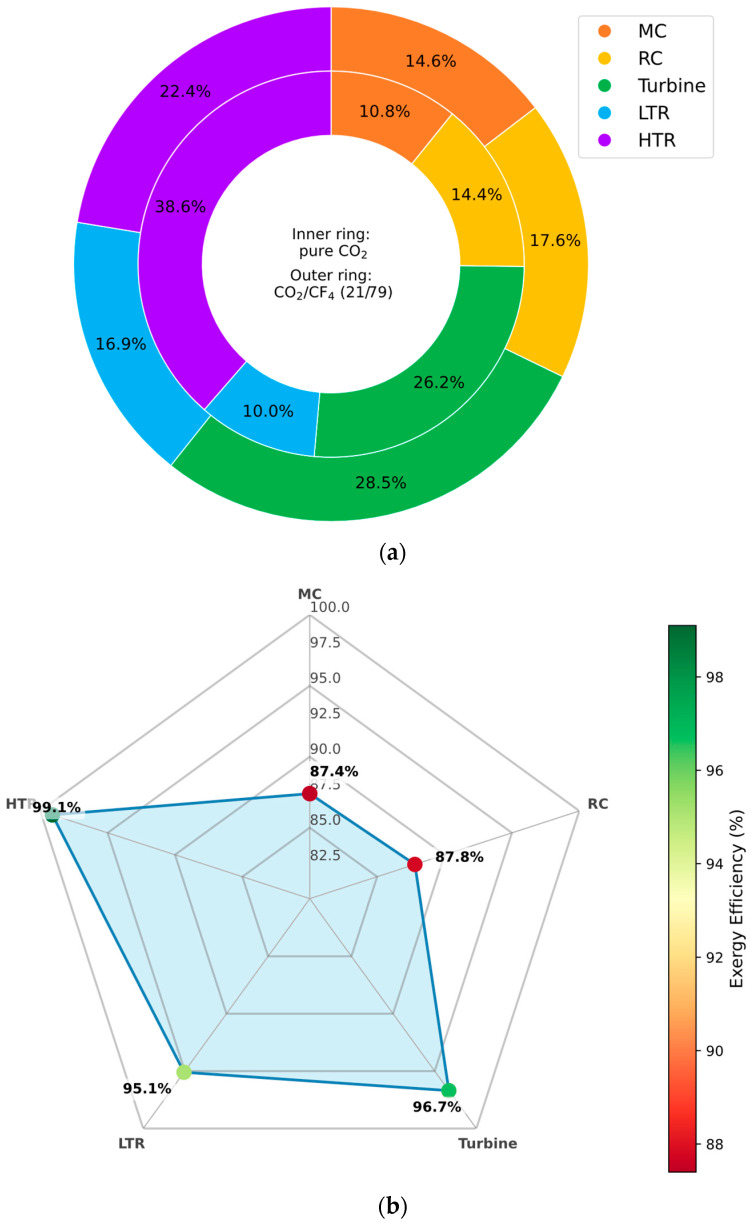
(**a**) Exergy destruction and (**b**) Exergy efficiency with CO_2_/CF_4_ mixture.

Figure 13 presents a thermodynamic evaluation of the recompression Brayton cycle components when operating with a CO_2_/CF_4_ mixture as the working fluid. Specifically, Figure 13a compares the percentage distribution of exergy destruction between the baseline pure CO_2_ case and the CF_4_-enriched mixture. The results indicate that exergy destruction is notably reduced in the HTR when the mixture is employed, highlighting improved thermal recovery and reduced irreversibility in this component.

Furthermore, Figure 13b reveals a corresponding increase in exergy efficiency, not only in the HTR but also in the turbine. This improvement can be attributed to the smoother thermodynamic behavior of the CO_2_/CF_4_ mixture at low temperatures, which enhances expansion characteristics and reduces entropy generation during the heat recuperation and expansion processes. These findings reinforce the value of CF_4_-rich mixtures in improving component-level second law performance, particularly in cold-climate cycle configurations.

#### 3.6.3. CO_2_/CH_4_ Mixture

**Figure 14:**
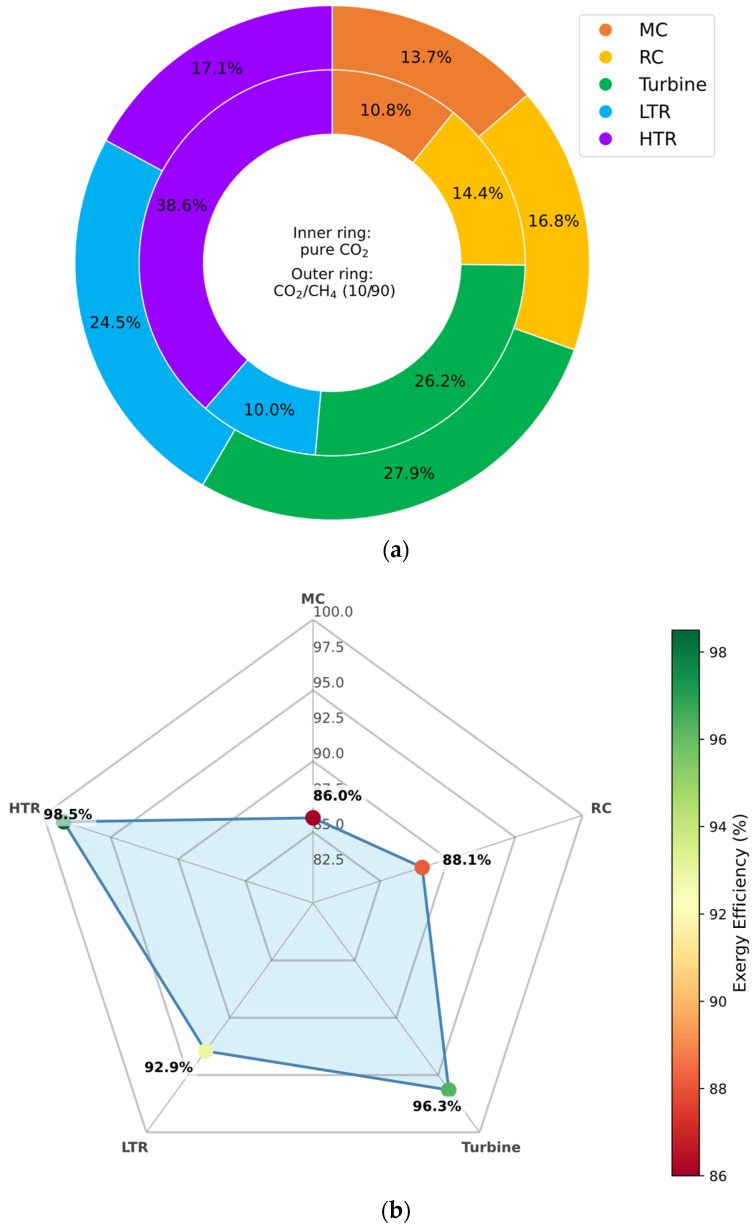
(**a**) Exergy destruction and (**b**) exergy efficiency with the CO_2_/CH_4_ mixture.

As illustrated in Figure 14a, the use of CO_2_/CH_4_ mixtures leads to increased exergy destruction across all major cycle components, with the exception of the high-temperature recuperator (HTR). Notably, the HTR displays a lower exergy destruction rate in the CH_4_-rich configuration compared to the pure CO_2_ baseline. However, this improvement is partially offset by a decrease in the HTR’s overall exergy efficiency—approximately 11% lower—suggesting a higher thermal duty requirement to achieve similar effectiveness under altered fluid conditions.

Figure 14b further shows that exergy efficiency decreases in all components except for the HTR, which registers a marginal improvement of approximately 1%. This slight gain likely results from the modified heat transfer behavior of CH_4_-rich mixtures in the high-temperature region, which promotes more favorable entropy generation dynamics in the HTR segment.

#### 3.6.4. CO_2_/NF_3_ Mixture

In the case of the CO_2_/NF_3_ mixture, the exergy analysis reveals a notable reduction in irreversibility within the high-temperature recuperator (HTR), with exergy destruction approximately 17.5% lower than in the pure CO_2_ configuration. The turbine also exhibits a favorable performance shift, registering a 6.5% reduction in exergy destruction.

**Figure 15:**
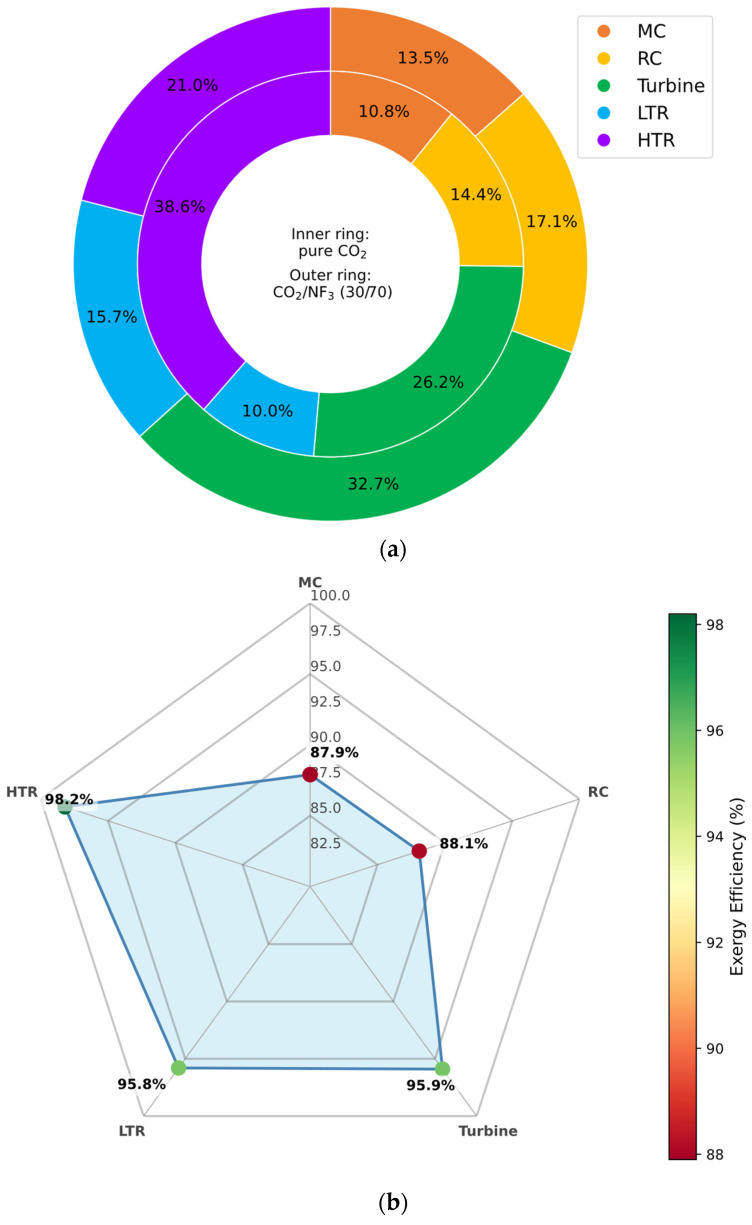
(**a**) Exergy destruction and (**b**) exergy efficiency with the CO_2_/NF_3_ mixture.

However, as illustrated in Figure 15a, detailed evaluation of component-wise exergy efficiency shows only a marginal improvement in the HTR relative to the baseline. This indicates that although total exergy losses are reduced, the ratio of useful exergy recovered to the total available exergy remains nearly unchanged—suggesting that the benefit stems more from improved fluid thermophysical behavior than from enhanced component effectiveness per se.

These results position CO_2_/NF_3_ mixtures as thermodynamically advantageous in reducing overall irreversibilities, especially in heat recuperation and expansion stages, while maintaining performance stability in the face of moderate ambient temperature reductions.

#### 3.6.5. CO_2_/Kr Mixture

**Figure 16:**
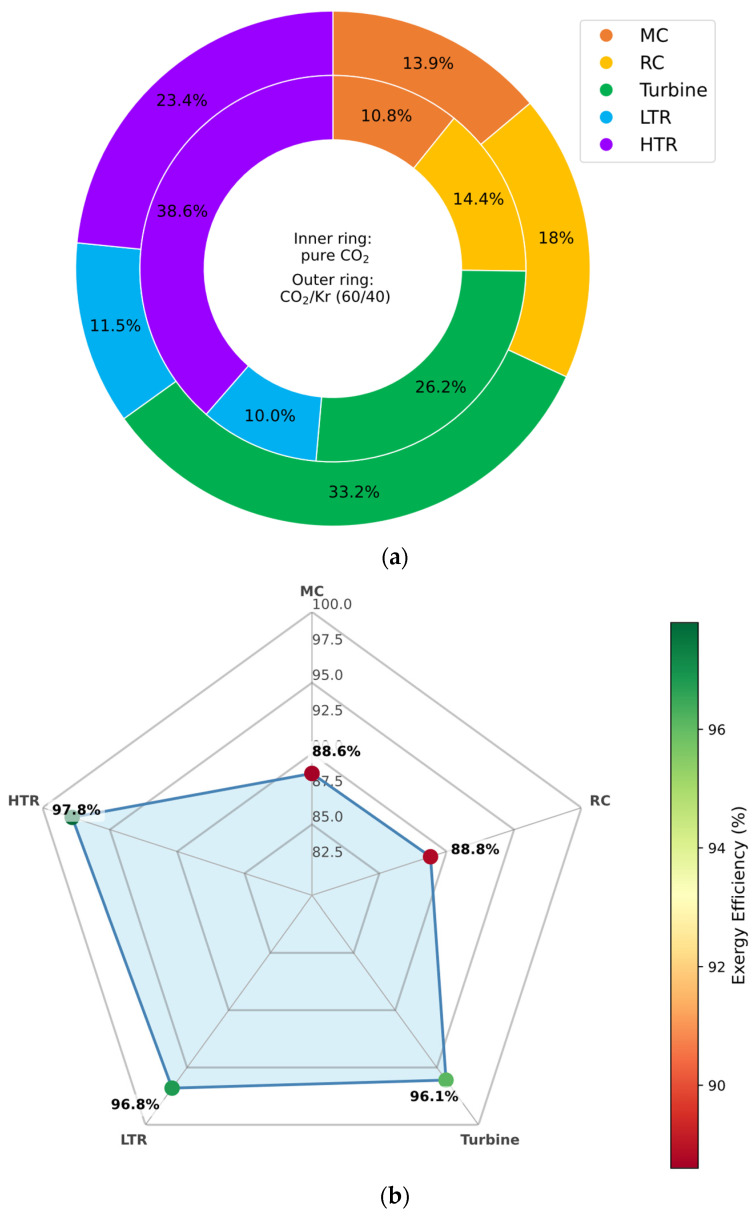
(**a**) Exergy destruction and (**b**) exergy efficiency with the CO_2_/Kr mixture.

The CO_2_/Kr mixture follows the general exergy trend observed across the studied binary blends. As shown in Figure 16, exergy destruction is reduced in all major cycle components relative to the pure CO_2_ baseline, with the exception of the high-temperature recuperator (HTR), where irreversibility remains largely unchanged.

Notably, marginal improvements in exergy efficiency are observed in the main compressor (MC) and the HTR. These gains can be attributed to krypton’s high molar mass and favorable thermophysical properties, which contribute to more stable flow characteristics and reduced entropy generation in compression and heat recovery stages. However, the limited thermodynamic enhancement of Kr-rich mixtures—reflected in their more modest efficiency improvements—results in a flatter exergy efficiency profile compared to more aggressive additives like CF_4_ or CH_4_.

As summarized in Table 4 and based on Equation (33), a comparative analysis is presented encompassing key performance indicators for the recompression Brayton cycle using the various working fluid mixtures examined in this study. The parameters include thermal efficiency, equivalent Carnot efficiency, the distribution of component-level irreversibilities, and total entropy generation. This integrated evaluation provides a comprehensive overview of the second-law performance across the studied fluid configurations, highlighting the trade-offs and synergies associated with each mixture under cold-climate operating conditions.

## 4. Conclusions

Blending CO_2_ with low-critical-temperature additives significantly enhances the thermodynamic performance of supercritical CO_2_ (s-CO_2_) recompression Brayton cycles under cold ambient conditions. The binary mixtures evaluated—CO_2_ with CF_4_, CH_4_, NF_3_, and Kr—demonstrated superior performance over pure CO_2_ in terms of higher thermal efficiency, lower specific compression work, larger turbine expansion ratios, and improved heat recuperation, particularly at the cold end of the cycle.

By lowering the critical point of the working fluid, these mixtures enable stable supercritical operation at subzero compressor inlet temperatures (CIT), eliminating two-phase instabilities and allowing deeper expansion in the turbine. These effects result in net thermal efficiency gains of several percentage points. Notably, at ambient temperatures between −40 °C and −50 °C, CO_2_/CF_4_, CO_2_/CH_4_, and CO_2_/NF_3_ blends delivered improvements of up to 14 percentage points over the pure CO_2_ cycle, consistent with the trend that greater deviation from CO_2_’s critical temperature increases the value of an optimized working fluid blend.

The degree of performance improvement was found to depend heavily on both fluid composition and heat exchanger sizing. In general, increasing the additive concentration (typically within 50–90 mol%) depressed the critical point sufficiently to preserve supercritical operation at very low temperatures, effectively mitigating condensation at the compressor inlet. While Kr mixtures, such as 20% CO_2_/80% Kr, yielded modest improvements (~53% efficiency), their benefits were limited compared to CF_4_, CH_4_, and NF_3_ mixtures, which achieved 7–10% efficiency gains within similar concentration ranges. These mixtures not only improved phase stability but also enhanced recuperator performance due to altered heat capacity profiles and wider temperature differentials.

Thermal performance enhancements were strongly linked to the recuperators’ total conductance (UA). Without sufficient UA, pinch-point limitations prevented full utilization of the improved fluid properties. The simulations showed that increasing UA, particularly in the high-temperature recuperator (HTR), was crucial for maximizing efficiency. Mixtures with high additive content generated larger heat recuperation duties and required correspondingly scaled heat exchangers to avoid thermal bottlenecks. Proper sizing—e.g., HTR UA > 5000 kW/K for CH_4_- and CF_4_-rich fluids—was essential to fully leverage their improved heat recovery characteristics.

Based on these findings, binary mixtures with 50–90 mol% of low-critical-temperature additives are recommended for s-CO_2_ cycles intended for subzero environments. Mixtures such as CO_2_/CF_4_ at 30/70 and CO_2_/NF_3_ at 20/80 enable consistent single-phase supercritical operation while minimizing compression work and enhancing turbine performance. CF_4_-containing blends, in particular, exhibited increasing thermal efficiencies (up to 62%) with mole fraction, outperforming the other mixtures across most operating scenarios.

Additionally, the exergy efficiency analysis indicated that most cycle components exhibited similar performance when operating with mixtures. However, the HTR consistently showed the highest exergy efficiency across all configurations, reaching 99.1% in the CO_2_/CF_4_ case, underscoring its critical role in cycle performance.

Design Recommendations:

(1) Working Fluid Selection

Binary CO_2_-based mixtures with 50–90 mol% of CF_4_, CH_4_, or NF_3_ are recommended for cold-climate s-CO_2_ cycles, ensuring supercritical operation, reduced compressor work, and increased turbine expansion ratios at ambient temperatures down to −50 °C.

(2) Heat Exchanger Design Optimization

The low- and high-temperature recuperators must be resized, particularly the HTR, to accommodate increased heat recovery loads. For CH_4_- and CF_4_-rich mixtures, HTR UA values exceeding 5000 kW/K are advised to prevent pinch-point constraints.

(3) System-Level Integration Considerations

Although CH_4_ mixtures yield the highest efficiencies, their associated high volumetric flow rates and turbomachinery scaling requirements necessitate thorough techno-economic evaluation. CF_4_ and NF_3_ mixtures present more favorable integration profiles, facilitating retrofitting of existing s-CO_2_ systems.

(4) Environmental and Economic Considerations

Due to the high global warming potential (GWP) of CF_4_ and NF_3_, future designs should pursue mixtures that balance performance with environmental impact. Lifecycle emissions, leakage mitigation, and alternative additives with lower GWP should be explored.

**Future work**

The promising results observed with binary mixtures suggest further performance improvements could be achieved through ternary formulations. Combining CO_2_ with a fluorinated additive (e.g., CF_4_ or NF_3_) and a benign, near-ideal gas (e.g., Ar, N_2_, Xe, CO) could yield mixtures with enhanced thermophysical properties and reduced environmental burden. Preliminary simulations indicate that ternary blends allow finer control over critical temperature, specific heat ratio, and fluid density—enabling higher efficiencies at extreme CITs (<−50 °C) while minimizing reliance on high-GWP compounds.

These three-component systems exploit the synergistic attributes of their constituents: high-density additives enable improved recuperation, while lighter inert gases increase stability and environmental compatibility. Certain ternary blends demonstrated superior performance to their binary counterparts in exploratory simulations. Future research should focus on optimizing ternary mixture design, validating their thermophysical properties experimentally, and assessing long-term operability—including materials compatibility and leakage risks—paving the way for next-generation cold-climate s-CO_2_ power systems.

## Figures and Tables

**Table 1:** Pure fluids which are used for mixing with s-CO_2_.

Working Fluid	Critical Temperature (K)	Critical Pressure (kPa)	Critical Density (Kg/m^3^)
CO_2_	304.13	7377.30	467.60
NF_3_	234.00	4460.70	562.47
CF_4_	227.51	3750.00	625.70
Kr	209.48	5525.00	909.21
CH_4_	190.56	4599.20	162.66

**Table 2:** Benchmarking and validation of the SCSP software.

Parameter	Reference Value [46]	Present Work’s Value	Error (%)
Power Output (MW)	10	10	---
T1 (K)	308.15	308.15	---
T2 (K)	346.15	346.18	0.01
T3 (K)	439.15	440.61	0.33
T4 (K)	No data	448.36	---
T5 (K)	812.15	812.66	0.06
T6 (K)	988.15	988.15	---
T7 (K)	861.15	861.05	0.01
T8 (K)	453.15	453.01	0.03
T9 (K)	351.15	351.21	0.02
T10 (K)	467.15	467.23	0.02
P1 (MPa)	8.50	8.50	---
P2 (MPa)	26.50	26.50	---
P3 (MPa)	26.20	26.20	---
P4 (MPa)	26.20	26.20	---
P5 (MPa)	25.80	25.80	---
P6 (MPa)	25.00	25.00	---
P7 (MPa)	9.20	9.20	---
P8 (MPa)	8.90	8.90	---
P9 (MPa)	8.70	8.70	---
P10 (MPa)	26.20	26.20	---

**Table 3:** Key parameters for the s-CO_2_ recompression Brayton cycle.

	Parameter	Value	Units
Net power output [46]	${\dot{W}}_{net}$	10	MW
Turbine inlet temperature [46]	TIT	988.15	K
Compressor inlet temperature *	CIT	≳$T_{cr}$	K
Compressor inlet pressure **	CIP	$P_{cr}+2$	MPa
Compressor outlet pressure [46]	$P_{MC,out}$	26.5	MPa
Heat exchange conductance for the LTR [46]	$UA_{LTR}$	1550 → 5250	kW/K
Heat exchange conductance for the HTR [46]	$UA_{HTR}$	1950 → 5500	kW/K
Turbine isentropic efficiency [47]	$\eta_{turb}$	0.85	--
Main compressor isentropic efficiency [47]	$\eta_{MC}$	0.80	--
Re-compressor isentropic efficiency	$\eta_{RC}$	0.71	--
Generator efficiency	$\eta_{g}$	0.96	--
Recompression Fraction	γ	optimized	--
Pressure drops for LT cold [48]	$\Delta P/P_{LT,cold}$	0.0113	--
Pressure drops for LT hot [48]	$\Delta P/P_{LT,hot}$	0.0225	--
Pressure drops for HT cold [48]	$\Delta P/P_{HT,cold}$	0.015285	--
Pressure drops for HT hot [48]	$\Delta P/P_{LT,hot}$	0.0326	--
Pressure drops for precooler [48]	$\Delta P/P_{PreC}$	0.023	--
Pressure drops for PHX [48]	$\Delta P/P_{PHX}$	0.031	--

* The CIT for the mixtures is just above the critical temperature. ** The CIP is 2 MPa above the critical pressure.

**Table 4:** Summary of the results obtained for the RBC.

Working Fluid	$\eta_{th}$	$\eta_{Eq-Carnot}$	$\sigma_{total}\left(\frac{kW}{K}\right)$
Pure CO_2_	0.4568	0.6322	12.39
CO_2_/CF_4_ (21/79)	0.5770	0.7228	10.15
CO_2_/CH_4_ (10/90)	0.5859	0.7389	11.77
CO_2_/NF_3_ (30/70)	0.5350	0.6992	11.38
CO_2_/Kr (60/40)	0.5125	0.6637	10.77

## Data Availability

Data will be made available on request.

## Nomenclature

Abbreviations	
CF_4_	Tetrafluoromethane
CH_4_	Methane
CIP	Compressor inlet pressure
CIT	Compressor inlet temperature
CO_2_	Carbon dioxide
CSP	Concentrated solar power
LT	Low temperature
h	Specific enthalpy [kJ/kg]
HT	High temperature
HTR	High temperature recuperator
Kr	Krypton
NF_3_	Nitrogen Trifluoride
MC	Main Compressor
${\dot{m}}_{comp}$	Mass flow of the compressor [kg/s]
${\dot{m}}_{turb}$	Mass flow of the turbine [kg/s]
NIST	National Institute of Standards and Technology
RBC	Recompression Brayton cycle
RC	Recompressor
LTR	Low temperature recuperator
PC	Pre-Compressor
PHX	Primary heat exchanger
$P_{MC,out}$	Compressor outlet pressure
PreC	Precooler
$\dot{Q}$	Heat transfer ratio
s	Specific entropy [kJ/kg·K]
s-CO_2_	Supercritical carbon dioxide
SCSP	Supercritical concentrated solar power plant
$T_{0}$	Ambient temperature [K]
$T_{abs}$	Absorption temperature [K]
$T_{rej}$	Rejection temperature [K]
$UA_{total}$	Heat total recuperator conductance [kW/K]
$\dot{W}$	Power [kW]
Greek Symbols	
$\eta_{Eq-Carnot}$	Equivalent Carnot efficiency
$\eta_{comp}$	Compressor efficiency
$\eta_{g}$	Generator efficiency
$\eta_{turb}$	Turbine efficiency
$\eta_{th}$	Thermal efficiency
$\eta_{ex}$	Exergy efficiency
$\dot{\sigma }$	Entropy generated [kW/K]
γ	Split flow ratio
